# Element Geochemical Characteristics and Geological Significance of Mudstones from the Middle Jurassic Shaximiao Formation in Sichuan Basin, Southwest China

**DOI:** 10.1021/acsomega.3c01496

**Published:** 2023-08-10

**Authors:** Di Ma, Zhijie Zhang, Chuanmin Zhou, Dawei Cheng, Haitao Hong, Hao Meng, Xinghe Yu, Zixiao Peng

**Affiliations:** † School of Energy Resources, China University of Geosciences, Beijing 100083, China; ‡ Research Institute of Petroleum Exploration and Development, Beijing 100083, China; § PetroChina Southwest Oil and Gas Field Company, Chengdu 610041, China

## Abstract

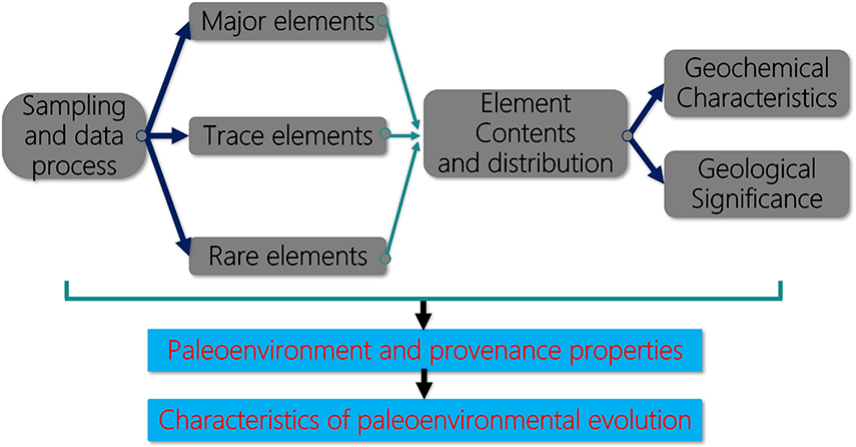

Thick sequences of terrestrial multicolored mudstones of the Middle Jurassic Shaximiao Formation in the Sichuan Basin, Southwest China, effectively recorded paleoclimate and paleoenvironment changes. The paleoenvironment of the Shaximiao Formation is reconstructed by using detailed sedimentological and elemental geochemical analysis of the multicolored mudstones. The provenance, paleoclimate, paleosalinity, and paleoredox conditions are distinguished by using the discriminant indicators of CIA, C-value, Sr/Cu, Rb/Sr, Th/U, V/Cr, and V/(V + Ni). The results show that all samples derive primarily from felsic igneous rocks and intermediate rocks rather than recycled sediments. The mudstone sequences were deposited under semiarid and semihumid regions with paleoclimate evolved to drier and cooler conditions from lower to upper Shaximiao Formation. Such a paleoclimate coincided with the records of several basins in the lower paleolatitudes of the Northern Hemisphere and were possibly affected by the Middle Jurassic global geological events such as wildfire, paleogeographic reorganizations, and seaway dynamics change. The paleowater body belongs to a typical terrestrial freshwater environment, although the paleosalinity increased significantly during arid periods. The multicolored mudstones were deposited in oxidation and weak-oxidation to weak-anoxic conditions. We also propose a detailed conceptual paleoenvironment model for Shaximiao Formation, with a large perennial lake surrounded by limited alluvial plain during a period of high lake level and small ephemeral lakes scattering extensive alluvial plain during a phase of low lake level.

## 1 Introduction

The element content of mudstone and the ratio of related elements in stratigraphic records are used to qualitatively and quantitatively reconstruct the paleoclimate and paleoenvironment.^1−3^ Chemical weathering caused by temperature and humidity changes, salinity changes in the environmental water body, and different redox conditions will result in the selective loss or enrichment of sensitive elements in sedimentary materials, which is essential for revealing the veil of paleoclimate and paleoenvironment conditions.^4−6^ Generally, the intensity of chemical weathering depends mainly on climate, and intensified chemical weathering occurs in warmer and wetter conditions. Climate-sensitive elements, such as Fe, Mn, Cr, V, Ni, and Co, are more readily enriched under humid climates, while in arid conditions, Ca, Mg, K, Na, Sr, and Ba are relatively enriched. Paleoclimatic conditions can be indirectly inferred from the ratio of various elements, such as Mg/Ca, Sr/Cu, and Th/U. CIA and C-values are also widely used as paleoclimate proxies.^7−9^ Salinity is an essential chemical feature of the water body, and elemental paleosalinity proxies, such as Sr/Ba, Rb/K, V/Ni, and Th/U are effective indices to judge the salinity of paleo-water body.^3,10^ In addition, trace elements can well reflect the redox conditions of the paleoenvironment. Previous studies have revealed that the Ni/Co, U/Th, V/(V + Ni), and V/Cr ratios could be used to reconstruct the paleoredox conditions.^1,11,12^

The Sichuan Basin, an intracontinental sedimentary basin in southwest China, rich in oil and gas resources, is widely famous for dinosaur research in the Middle Jurassic.^13^ The multicolored mudstone, Estherian fossil, and glauconite in sandstone preserved in the stratigraphic record are the result of the complex climate and environmental conditions that evolved during the Middle Jurassic. The element characteristics show that the western edge of the basin was warm and dry in the Middle Jurassic, and turned cool in local periods.^14^ The characteristics of clay minerals in the southwestern margin of the basin indicate a semi-arid climate dominated by intermittent cold and drought when Shaximiao Formation was deposited.^15^ The analysis of the paleosol and carbonate nodule in the northeast of the basin shows that this period is in the semiarid to semihumid climate and the dry-humid and cool/warm temperate climates alternately.^16^ These studies reveal the paleoclimate of each region of the basin but lack of comprehensive analysis combined with mudstone type, paleo-water body, and oxidation conditions.

In terms of the sedimentary environment, sedimentary indicators of the delta front, such as channel and mouth bars, have been found in cores in central Sichuan, while meandering river and floodplain deposits are dominant in outcrops in western and eastern Sichuan.^17^ Dark-gray and gray-green mudstones, rich in bivalves, and other paleontological fossil assemblages, indicate the existence of local large lakes during the flood/wet period in central and southern Sichuan, whereas purple-red soil layers and cross-bedded sandstone represent alluvial deposits during the dry period in the northeastern and western Sichuan Basin.^17,18^ Obviously, climate fluctuations have profoundly affected the distribution and evolution of the environment in the basin from the proximal to the distal domain, especially the size of the lake and the spatial arrange of the alluvial plain and lake, and changed the weathering intensity and paleoredox conditions of the basin, resulting in changes in the lithofacies of Shaximiao Formation. The existing research still fails to put forward an applicable environmental evolution model in combination with climate and sedimentary facies, which hinders the prediction and evaluation of tight sand reservoirs of Shaximiao Formation.

As the reconstruction of the paleoenvironment typically has been of particular benefit to promote the understanding of the distribution pattern of the sand body and predict the favorable lithologic reservoir, this paper describes the sedimentological characteristics of mudstone, emphatically systematically analyzes the geochemical characteristics of major elements, trace elements, and rare earth elements(REE) in mudstone, to reconstruct the paleoenvironment of the Shaximiao Formation by using applicability methods such as the ratio of each element to determine the paleoclimate, paleosalinity, paleoredox, and other environmental conditions. Furthermore, based on the characteristics of the paleoenvironment of the Sichuan Basin in this period, we propose an alluvial plain-lake model for the Shaximiao Formation.

## 2 Geological Setting

**Figure 1:**
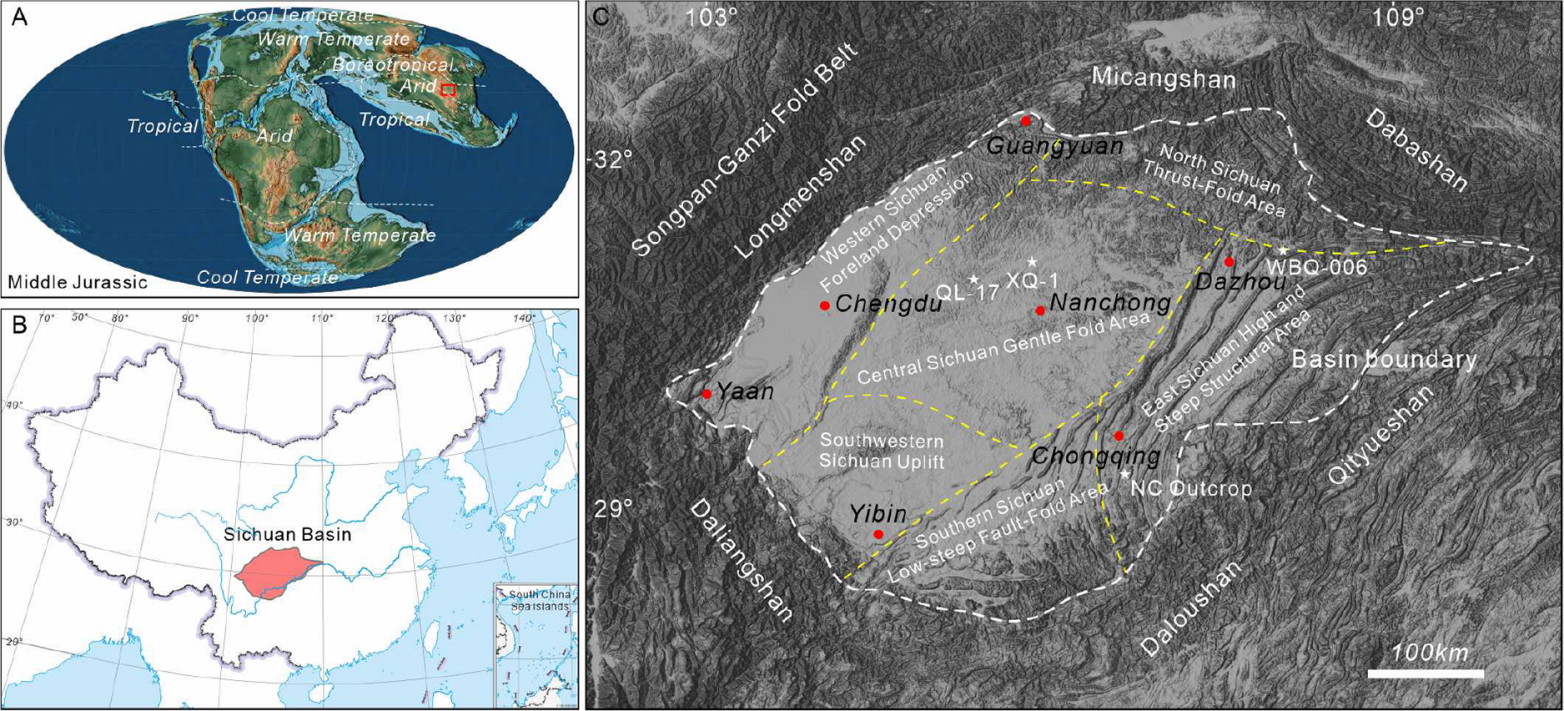
(A) Global climatic zones (Boucot et al., 2013) and paleogeographic map (Scotese, 2014) during the Middle Jurassic. The red rectangle represents the Sichuan Basin, which was in the boreotropical climate zone; (B) location of Sichuan Basin; (C) tectonic map of the Sichuan Basin showing the location of investigated wells and outcrops (well WBQ-006, QL-17, XC-1, and NC outcrop).

The Sichuan Basin, geographically located in the eastern part of Sichuan Province and the western part of Chongqing in southwest China, has an area of approximately 18 × 10^4^ km^2^ (Figure 1). The Sichuan Basin, which comprises the northwestern segment of the Yangtze Block in southern China, is located on the southern side of the Qinling orogenic belt, and at the intersection of the North China plate, the Yangtze plate, the Qinghai-Tibet Plateau, and other terranes.^19,20^ It is structurally located in the superimposed area of the Micangshan-Dabashan front thrust nappe belt and the eastern Sichuan arc-shaped fold belt. The basin has evolved from a passive continental margin to a foreland basin since the Middle Triassic, in response to multiphase collisions of the surrounding tectonic blocks.^21^ Considerable collisions between these blocks distinctly shaped the development from a shallow-marine carbonate platform to an intercontinental sedimentary basin.^22^ The Qinling orogen was thrust southwestward over the northeastern Sichuan basin along the Dabashan fold-and-thrust belts from the Middle Triassic to the Late Jurassic, forming the northeastern Sichuan foreland basin.^23,24^

**Figure 2:**
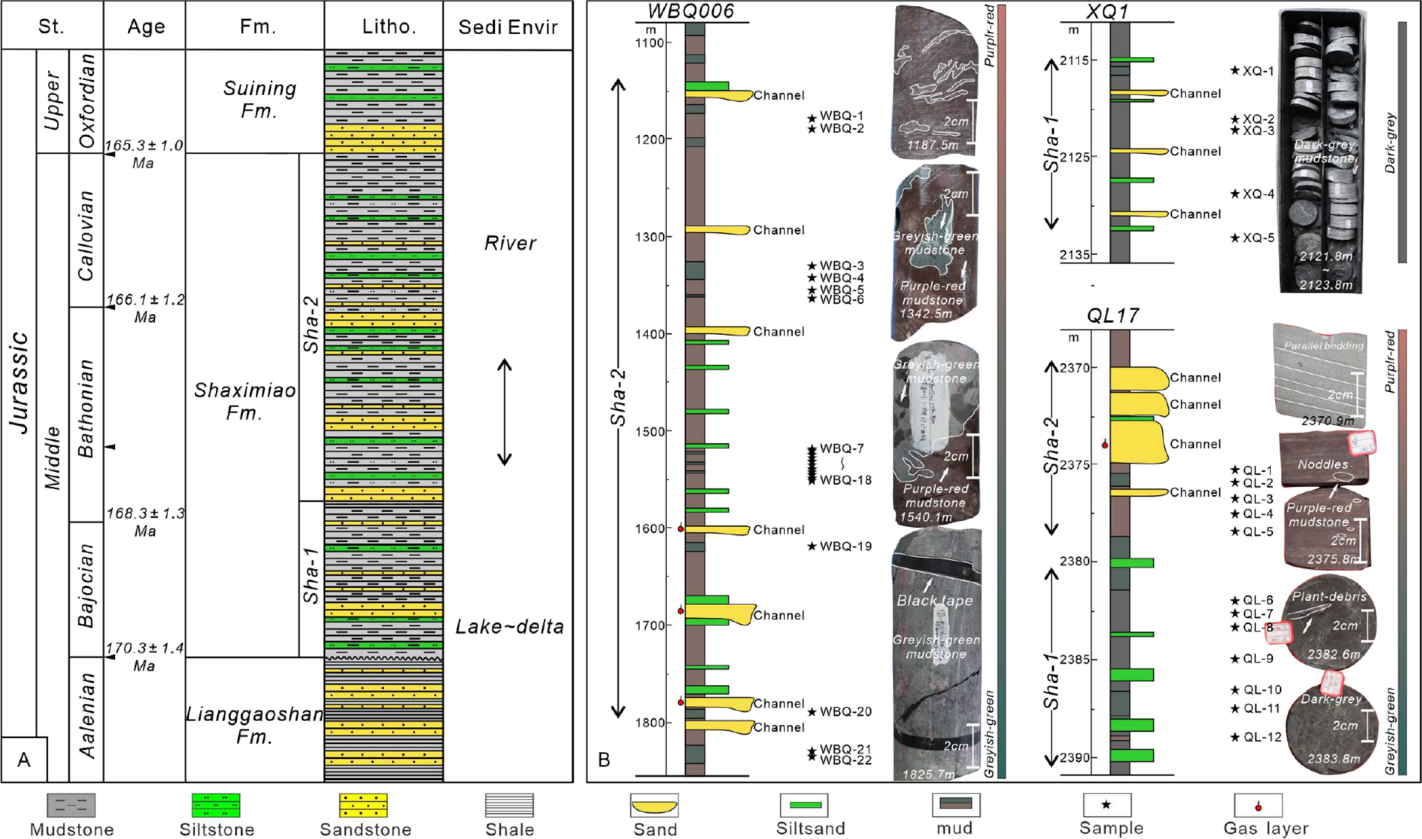
Stratigraphic column of the Shaximiao formation and the distribution of mudstone samples. (A)General stratigraphy of the Middle and Upper Jurassic for the Sichuan Basin with curves in last track showing globally averaged sea level change. (B) Sketch logs of wells analyzed showing distribution of core samples for chemical analysis. Systematic upward color changes in inserted core image reflect gradual changes in paleoclimate.

The northeastern Sichuan foreland basin experienced rapid flexural subsidence in the Middle and Late Jurassic, resulting in the deposition of thick- to medium-bedded sandstone interbedded with conglomerates. During the Middle Jurassic, the sedimentary stratigraphy distributed throughout the basin with thickness characterized by being thick in the northeast and thin toward the southwest. The Middle Jurassic strata in the basin consists of the Shaximiao Formation and the Lianggaoshan Formation (Figure 2A). The Lianggaoshangou Formation is mainly composed of purplish-red mudstone and siltstone with gray sandstone. The upper part conforms with the Suining Formation of the Upper Jurassic, and the lower part is in unconformity with the Shaximiao Formation. The Shaximiao Formation consists of purplish-red, gray-green mudstone and siltstone as well as interbedded gray fine- to medium-grained sandstone. According to well-developed paleosols, most of the mudstones and siltstones are interpreted to be deposits of alluvial plain.^23^ Indicated by trough cross-bedding, planar cross-bedding, parallel bedding, and massive bedding in sandbody, interbedded sandstones are generally interpreted to be channel deposits of a river or delta system.^17^

## 3 Samples and Methods

### 3.1 Samples

In this study, 61 samples were collected from well WBQ006 in northeast Sichuan, well QL17 in central Sichuan, well XQ1 in southwest Sichuan, and Nanchuan Outcrop in southeast Sichuan, including the lower Shaximiao Formation (Sha-1 Member) and upper Shaximiao Formation (Sha-2 Member) (Figures 1C and 2B). All samples were taken from purple-red, grayish-green, and dark-gray massive mudstone and analyzed for major, trace, and REE analysis. To avoid potential external pollution, we selected freshly exposed faces for sampling after chipping off the weathered/polluted surface of the sample. All samples were stored in plastic bags and put in the sample bag for standby. We recorded the depth, lithology, and sedimentary phenomenon of each sample and calibrated the core depth at the logging curve. The mudstone samples were provided by the Institute of Petroleum Exploration and Exploitation, PetroChina Southwest Oil and Gas Field Company. The sample pretreatment and geochemical experimental analysis were carried out at the Petroleum Geology Research and Laboratory Center, Research Institute of Petroleum Exploration and Development, China.

### 3.2 Testing Methods

The major elements were analyzed by an X-ray fluorescence spectrometer (XRF), including SiO_2_, Al_2_O_3_, CaO, K_2_O, Na_2_O, Fe_2_O_3_, MnO, MgO, TiO_2_, and P_2_O_5_. All mudstone samples were first crushed to 200 mesh (<40 μm). The samples of 1.2–1.5 g were accurately weighed and then heated at 105 ° C for 4 h. After cooling for 2 h, 50 ± 500 mg of samples, cosolvent (Li_2_B_4_O_7_, LiBO_2_, and LiF), and oxidant (NH_4_NO_3_) are mixed and heated at 1150–1250 ° C for 15 min. Finally, the prepared samples were analyzed in the X-ray fluorescence spectrometer under the conditions of 24 ° C and 38% relative humidity. The test uncertainty is generally less than 4% for all major elements.

Trace elements were measured by ICP-MS. 200 mesh mudstone samples (<40 μm) were dried in an oven at 105 ° C for 2–4 h. 50 mg of powder sample, 1 mL of high-purity HF, and 1 mL of high-purity HNO_3_ were then mixed and heated in an oven at 190 ° C for more than 24 h. After cooling and drying with an electric heating plate, each sample was mixed with 1 mL of HNO_3_ and dried again. Then, each sample was mixed with 5 mL of HNO_3_ and heated at 190 ° C for 12 h. Finally, the prepared samples were analyzed in the ICP-MS at 23.6 ° C and 42% relative humidity. Analytical precision and accuracy are generally better than 5% for most trace elements.

### 3.3 Enrichment Factors

The geochemical properties of elements are influenced not only by their nature but also by the sedimentary environment and sedimentary process. Therefore, some elements have dispersion and enrichment differences in a special sedimentary environment. To use the element concentrations to reconstruct the paleoenvironment, it is necessary to evaluate whether they are relatively enriched or depleted. The element enrichment factor (EF) can reflect the enrichment or loss of the relative content of elements in the sediment, defined as follows:
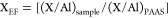
where X and Al represent respectively the content of trace element and aluminum in the sediments. The samples are usually normalized using the composition of the post-Archean Australian shale (PAAS) from Taylor and McLennan (1985).^25^ X_EF_ > 1 indicates that the sample X element is enriched relative to the average shale, X_EF_ > 10 indicates that the sample X element is strongly enriched, and X_EF_ < 1 indicates that the sample X element is relatively deficient. This method is used to evaluate the enrichment degree of major and trace elements in mudstone samples from the Shaximiao Formation.

### 3.4 Paleoweathering Indices

Several researchers have utilized the weathering index of ancient detrital sediments to trace changes in chemical weathering. Different degrees of chemical weathering occurs in moist or arid environments due to differences in temperature or humidity acting on patent rocks.^2,6^ Hence, the Chemical Index of Alteration (CIA )values represent degrees of chemical weathering and have been widely used to reconstruct the paleoclimate and weathering intensity. The CIA value was calculated using molecular proportions in mudrocks^2^ as follows:
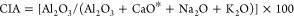
where CaO* represents CaO in the silicate fraction only. The correction methods for CaO* follow those described by McLennan.^6^ High CIA values indicate the leaching of the alkali elements and the relative stability of residual cations (Al^3+^, Si^4+^, and Ti^4+^). On the contrary, the low CIA value reflects that the composition of clastic material experiences fewer chemical changes. Hence, unweathered crystalline source rocks are characterized by a low CIA value (less than 55).^26^ With the gradual weathering and loss of mobile elements, the value is close to 100. The average CIA values for shales range from 70 to 75. In hot and humid tropical conditions, sediments have a high CIA value (85∼100), and the sediments deposited in the warm and humid paleoclimate have a medium CIA value (70∼85), while the lower CIA value (50–70) represents the cold and dry paleoclimate.

The paleoclimate index C-value is used as an indicator of paleoclimate, defined as follows:^27,28^
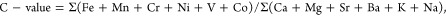
where elemental concentrations are presented as ppm. The C-value may be used to evaluate the changes in paleoclimate from warm and humid to hot and arid.^29^ 0 < C-value < 0.2 indicates an arid environment, 0.2 < C-value < 0.6 indicates a semi-arid to semi-humid environment, and C-value > 0.6 indicates a humid environment.^30,31^

## 4 Results

### 4.1 Sedimentary Characteristics of Mudstone

The origin and cause of the colors in the mudstone are generally emphasized, including the implications of environmental, climatic, and diagenetic factors.^32,33^ Field observation of outcrops and cores from the Shaximiao Formation shows that the color of mudstone is mainly purplish-red, grayish-green, and locally dark gray. The strong vertical heterogeneity of color indicates that the sedimentary environment changes frequently on the surface. The mudstone of the Shaximiao Formation is divided into purplish-red mudstone lithofacies (PRM), gray-green mudstone lithofacies(GGM), and dark-gray mudstone lithofacies(DGM) according to fresh surface color. We have calculated the fraction of each color facies in the Sha-1 and Sha-2 Member, respectively.

**Figure 3:**
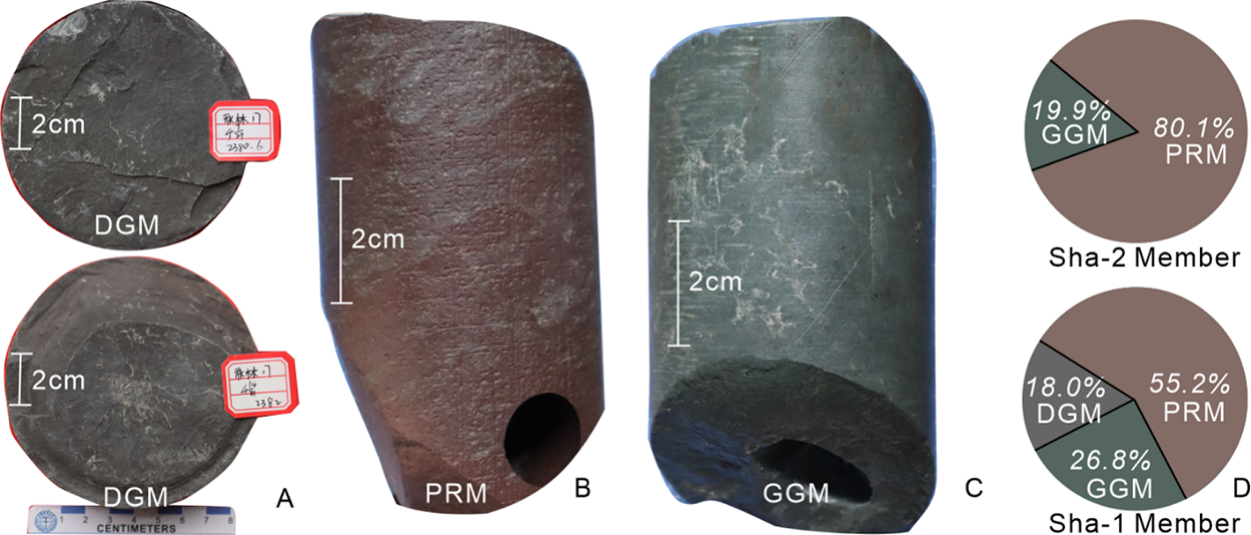
Types of mudstone lithofacies and their proportions in the Shaximiao Formation.

DGM consist mainly of shale and laminated mudstone. Minute plant debris can be observed in all gray mudstones and is the chief coloring agent (Figure 3A). Plant fragments are common and increase in abundance in darker mudstone, resulting in a higher fraction of organic matter in DGM than PGM and GGM. This color, which results in an anoxic deposition condition, typically indicates a water body with a certain depth that has been stable for some time.^34^ DGM is only visible at the top of Sha-1 Member, accounting for about 18% (Figure 3D).

PRM is the main mudstone color, characterized by rapid physical weathering and slow chemical pedogenetic processes (Figure 3B). Purple color is considered to be a function of the amount of hematite present related to oxidation.^33,35^ PRM comprises 55.2% of Sha-1 members and 80.1% of Sha-2 members (Figure 3D).

GGM is predominantly composed of claystone and mudstone. Most green rocks are uniformly colored throughout, even when in abruptly contact with PRF (Figure 3C). Green colors are deriving from the ferrous iron in chlorite and illite and indirectly to the lack of other coloring agents, representing the product of a hypoxic environment.^36^ These lithofacies accounts for 26.8% of Sha-1 Member and 19.9% of Sha-2 Member (Figure 3D).

### 4.2 Major Element Geochemistry

**Figure 4:**
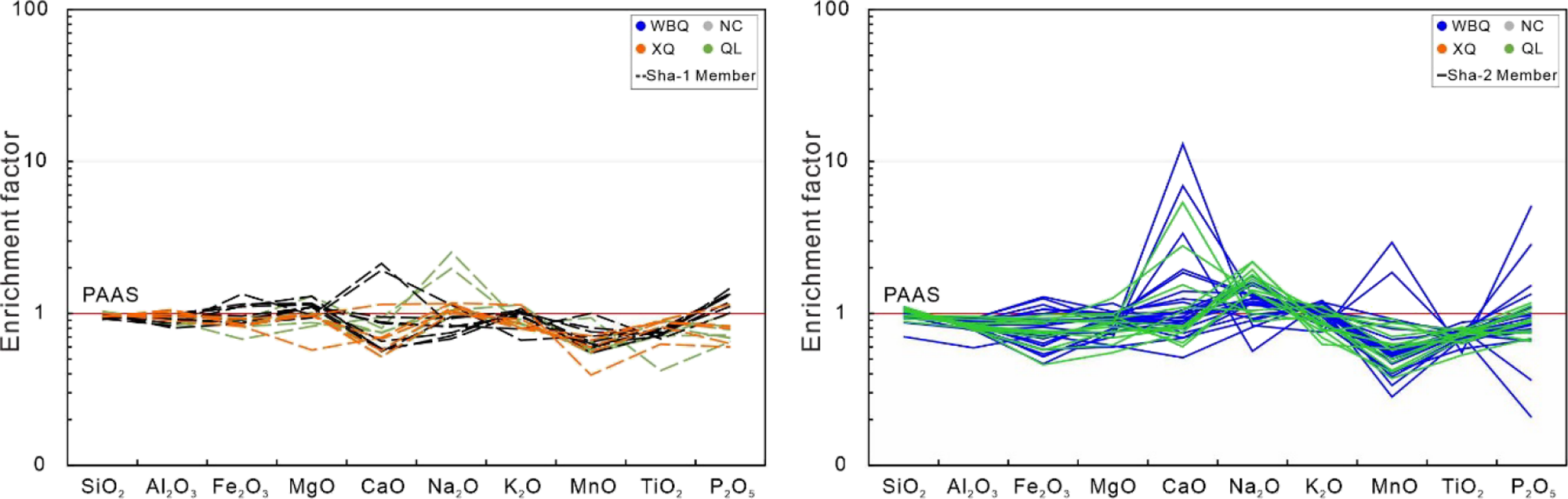
Enrichment factor of the major elements relative to the PAAS.

Results of major elements (oxides in wt %) are listed in Table 1, comparing North American Shale Composite (NASC) and Post-Archean Australian Shale (PAAS).^25,37^ The main element oxides SiO_2_ and Al_2_O_3_ in mudstone of the Shaximiao Formation are 44.1–76.49% and 11.23–19.96% respectively, with an average content of 61.97% and 16.64%, followed by CaO (0.66–8.97; average = 1.70), MgO (1.21–2.76; average = 2.02), Fe_2_O_3_ (2.76–9.28; average = 6.04), K_2_O (1.21–4.49; average = 3.36), and Na_2_O (0.85–3.97; average = 1.56). It also contains a small amount of MnO (0.03–0.32; average = 0.07), P_2_O_5_ (0.03–0.82; average = 0.16), and TiO_2_ (0.42–0.90; average = 0.73). A plot of the major elements, normalized to Post-Archean Australian shale (PAAS), shows that the SiO_2_, Al_2_O_3_, Fe_2_O_3_, MgO, MnO, TiO_2_, and K_2_O are slightly depleted whereas other elements show relative enrichment (Figure 4). The SiO_2_/Al_2_O_3_ values vary between 2.97 and 6.57, with an average of 3.93 (Table 1), indicating that the clay mineralogy of mudstone mainly consists of smectite. The K_2_O/Al_2_O_3_ ratios in the Shaximiao mudstone (Table 1) range from 0.10 to 0.30 (average = 0.20), which are consistent with PAAS. In addition, major elements of Sha-1 and Sha-2 Member show obvious similarity. The commonly used indicators of paleoclimate based on major elements include C-value, CIA, and T also, which are provided in Table 1.

**Table 1:** Major Element Content Data of Shaximiao Formation Mudstone

member	sample	mudstone litho.	SiO_2_	Al_2_O_3_	Fe_2_O_3_	MgO	CaO	Na_2_O	K_2_O	MnO	TiO_2_	P_2_O_5_	SiO_2_/Al_2_O_3_	Al_2_O_3_/TiO_2_	TiO_2_/Zr	K_2_O/Al_2_O_3_	CIA	C-value	*T*
Sha-2	WBQ-1	PRM	54.53	14.85	3.90	1.54	8.97	1.57	3.06	0.21	0.64	0.14	3.67	23.35	5.73	0.21	70.55	0.17	13.81
Sha-2	WBQ-2	PRM	58.67	17.49	9.05	1.94	0.90	1.08	4.30	0.06	0.70	0.12	3.35	24.88	4.48	0.25	73.03	0.26	15.20
Sha-2	WBQ-3	PRM	62.19	17.36	6.52	1.99	1.11	1.86	3.66	0.07	0.75	0.18	3.58	23.27	4.01	0.21	70.17	0.23	13.60
Sha-2	WBQ-4	GGM	65.71	16.06	4.90	2.02	1.16	1.50	3.79	0.05	0.76	0.16	4.09	21.24	7.13	0.24	70.28	0.33	13.66
Sha-2	WBQ-5	PRM	62.00	16.11	7.68	2.11	1.21	1.44	3.99	0.06	0.75	0.25	3.85	21.57	6.98	0.25	70.10	0.31	13.56
Sha-2	WBQ-6	PRM	62.51	15.66	6.19	2.09	2.41	1.55	3.68	0.09	0.74	0.18	3.99	21.11	7.20	0.23	69.79	0.36	13.38
Sha-2	WBQ-7	PRM	64.86	15.91	4.58	2.09	1.82	1.62	3.83	0.07	0.74	0.15	4.08	21.62	7.29	0.24	69.23	0.36	13.07
Sha-2	WBQ-8	GGM	63.63	15.88	4.40	2.09	2.53	1.58	3.84	0.10	0.77	0.16	4.01	20.54	7.09	0.24	69.41	0.38	13.17
Sha-2	WBQ-9	PRM	61.06	16.26	7.70	2.21	1.62	1.36	3.95	0.06	0.78	0.21	3.76	20.74	7.00	0.24	70.91	0.34	14.01
Sha-2	WBQ-10	PRM	67.26	15.88	3.74	1.75	1.24	1.87	3.67	0.04	0.68	0.13	4.24	23.53	7.05	0.23	68.18	0.45	12.48
Sha-2	WBQ-11	GGM	57.72	17.42	9.28	2.26	1.54	1.11	4.44	0.06	0.72	0.46	3.31	24.36	6.44	0.25	72.34	0.30	14.81
Sha-2	WBQ-12	PRM	62.53	16.85	6.43	2.04	1.30	1.50	4.08	0.05	0.73	0.14	3.71	23.11	5.74	0.24	70.41	0.26	13.73
Sha-2	WBQ-13	PRM	67.40	15.73	3.76	1.76	1.37	1.91	3.51	0.04	0.69	0.13	4.28	22.67	6.25	0.22	68.21	0.31	12.50
Sha-2	WBQ-14	GGM	69.69	14.73	3.36	1.67	1.16	2.11	3.19	0.05	0.58	0.11	4.73	25.27	6.92	0.22	66.53	0.35	11.56
Sha-2	WBQ-15	GGM	65.59	16.16	4.39	2.20	1.31	1.83	3.76	0.06	0.76	0.14	4.06	21.38	7.61	0.23	68.53	0.31	12.68
Sha-2	WBQ-16	PRM	54.10	11.23	5.23	1.67	6.92	1.00	2.78	0.32	0.55	0.82	4.82	20.34	7.25	0.25	70.14	0.34	13.58
Sha-2	WBQ-17	GGM	66.87	15.07	5.18	1.35	0.66	0.98	4.49	0.04	0.72	0.03	4.44	20.84	5.56	0.30	70.05	0.20	13.53
Sha-2	WBQ-18	GGM	60.15	14.59	5.86	1.60	4.36	0.68	4.38	0.10	0.73	0.11	4.12	19.99	6.29	0.30	71.79	0.25	14.50
Sha-2	WBQ-19	PRM	68.26	14.95	4.17	1.32	0.89	1.40	4.08	0.03	0.69	0.12	4.57	21.79	5.62	0.27	68.48	0.21	12.65
Sha-2	WBQ-20	GGM	61.69	16.76	8.23	1.81	1.03	1.39	3.26	0.06	0.79	0.06	3.68	21.22	6.48	0.19	73.51	0.36	15.46
Sha-2	WBQ-21	GGM	60.25	17.54	7.19	2.56	0.96	1.18	3.99	0.07	0.87	0.16	3.44	20.11	7.72	0.23	73.42	0.30	15.42
Sha-2	WBQ-22	GGM	63.92	16.32	5.98	2.08	1.16	1.40	3.66	0.06	0.79	0.12	3.92	20.79	5.99	0.22	71.64	0.27	14.42
Sha-2	NC-1	PRM	61.10	17.92	6.39	1.92	0.83	1.66	3.10	0.07	0.81	0.10	3.41	22.10	4.01	0.17	73.62	0.24	15.53
Sha-2	NC-2	PRM	68.66	14.63	4.13	1.68	1.03	2.32	2.30	0.06	0.74	0.14	4.69	19.69	3.18	0.16	67.83	0.34	12.28
Sha-2	NC-3	PRM	65.17	15.63	5.52	1.65	1.07	1.88	3.17	0.06	0.75	0.12	4.17	20.95	4.12	0.20	69.28	0.07	13.10
Sha-2	NC-4	PRM	69.42	15.24	3.31	1.21	0.97	2.62	2.89	0.04	0.53	0.11	4.56	28.81	3.27	0.19	65.21	0.23	10.82
Sha-2	NC-5	PRM	60.32	17.99	6.71	2.13	0.78	1.70	4.04	0.08	0.80	0.11	3.35	22.54	3.73	0.22	70.74	0.27	13.92
Sha-2	NC-6	PRM	64.53	15.94	5.35	1.79	0.97	2.12	3.71	0.09	0.71	0.18	4.05	22.55	4.09	0.23	66.72	0.23	11.66
Sha-2	NC-7	PRM	68.44	14.75	4.16	1.36	1.35	2.61	2.92	0.07	0.65	0.14	4.64	22.55	3.74	0.20	64.44	0.18	10.39
Sha-2	NC-8	PRM	55.21	15.03	5.24	1.88	6.99	1.06	3.54	0.10	0.63	0.15	3.67	23.97	3.92	0.24	72.64	0.22	14.98
Sha-2	NC-9	PRM	60.08	17.21	6.34	2.01	1.42	1.25	4.13	0.06	0.70	0.17	3.49	24.55	3.98	0.24	72.19	0.23	14.73
Sha-2	NC-10	PRM	66.87	14.78	5.00	1.94	1.07	2.15	2.55	0.05	0.66	0.14	4.52	22.29	3.44	0.17	68.33	0.29	12.57
Sha-2	NC-11	PRM	65.68	15.29	5.37	1.52	1.04	2.01	3.34	0.05	0.72	0.12	4.30	21.24	3.30	0.22	67.51	0.26	12.10
Sha-2	NC-12	PRM	58.13	16.26	6.17	2.76	3.61	1.66	2.81	0.10	0.77	0.19	3.58	21.03	4.34	0.17	72.62	0.35	14.97
Sha-2	NC-13	PRM	61.36	15.53	7.06	2.18	2.00	1.20	3.25	0.05	0.76	0.16	3.95	20.49	4.21	0.21	73.32	0.37	15.36
Sha-2	QL-1	GGM	70.01	12.43	3.70	1.31	3.08	2.96	1.87	0.10	0.46	0.13	5.63	26.90	2.92	0.15	61.47	0.25	8.73
Sha-2	QL-2	GGM	72.51	13.05	3.80	1.24	1.03	3.42	1.81	0.06	0.52	0.12	5.56	24.95	3.13	0.14	60.14	0.26	7.98
Sha-2	QL-3	PRM	76.49	11.65	2.76	0.82	0.92	3.97	1.21	0.04	0.38	0.08	6.57	31.07	2.14	0.10	56.01	0.27	5.67
Sha-2	QL-4	PRM	73.32	12.92	3.50	1.18	0.94	3.61	1.73	0.06	0.43	0.10	5.67	29.98	2.44	0.13	59.08	0.25	7.38
Sha-2	QL-5	PRM	62.29	11.94	3.85	1.37	7.58	2.63	1.90	0.25	0.58	0.20	5.22	20.48	3.13	0.16	62.51	0.26	9.31
Sha-1	XQ-1	GGM	62.91	16.85	5.94	1.26	0.90	1.40	4.19	0.04	0.63	0.10	3.73	26.96	4.70	0.25	70.68	0.16	13.88
Sha-1	XQ-2	DGM	60.18	18.65	6.65	2.11	0.88	1.25	2.92	0.07	0.89	0.18	3.23	20.88	5.99	0.16	77.48	0.34	17.69
Sha-1	XQ-3	DGM	59.34	19.94	5.93	2.16	0.67	1.20	3.37	0.06	0.82	0.10	2.98	24.35	5.46	0.17	77.56	0.34	17.73
Sha-1	XQ-4	DGM	60.13	17.94	6.62	2.14	1.49	1.39	2.98	0.08	0.88	0.13	3.35	20.32	5.70	0.17	75.70	0.39	16.69
Sha-1	XQ-5	DGM	59.66	19.50	6.10	2.12	0.74	1.31	3.17	0.07	0.87	0.13	3.06	22.39	5.12	0.16	77.11	0.29	17.48
Sha-1	NC-14	DGM	58.82	17.38	6.24	2.41	1.14	0.98	3.57	0.07	0.77	0.21	3.38	22.51	4.31	0.21	75.88	0.39	16.79
Sha-1	NC-15	DGM	58.74	18.87	6.94	2.41	0.74	0.85	3.97	0.06	0.72	0.19	3.11	26.06	4.50	0.21	76.87	0.40	17.35
Sha-1	NC-16	DGM	59.02	17.30	6.25	2.44	1.12	0.98	3.49	0.07	0.77	0.21	3.41	22.47	4.38	0.20	76.06	0.38	16.89
Sha-1	NC-17	DGM	57.74	15.67	9.65	2.14	2.76	0.99	2.91	0.11	0.68	0.16	3.68	23.08	3.92	0.19	76.19	0.39	16.96
Sha-1	NC-18	DGM	62.36	15.16	6.16	2.05	2.50	1.36	2.45	0.08	0.75	0.21	4.11	20.16	3.60	0.16	74.57	0.42	16.06
Sha-1	NC-19	DGM	58.59	18.30	7.99	2.51	0.76	0.81	3.73	0.07	0.71	0.23	3.20	25.81	4.57	0.20	77.35	0.39	17.62
Sha-1	NC-20	DGM	57.04	18.65	8.31	2.56	0.85	0.90	3.80	0.08	0.73	0.21	3.06	25.65	4.38	0.20	76.94	0.41	17.39
Sha-1	NC-21	DGM	58.84	18.01	6.55	2.42	1.23	1.11	3.86	0.07	0.79	0.22	3.27	22.74	4.60	0.21	74.76	0.33	16.17
Sha-1	NC-22	GGM	60.49	16.27	8.04	2.84	0.73	1.14	3.67	0.09	0.74	0.18	3.72	21.90	4.82	0.23	73.22	0.30	15.30
Sha-1	QL-6	DGM	60.84	18.27	6.51	2.22	0.97	1.37	2.97	0.07	0.89	0.13	3.33	20.44	4.65	0.16	76.19	0.27	16.97
Sha-1	QL-7	DGM	59.43	19.52	6.26	2.20	0.75	1.25	3.14	0.06	0.88	0.13	3.04	22.08	4.58	0.16	77.58	0.24	17.75
Sha-1	QL-8	DGM	59.25	19.96	6.01	2.20	0.68	1.15	3.37	0.06	0.82	0.11	2.97	24.34	4.62	0.17	77.88	0.30	17.91
Sha-1	QL-9	DGM	60.02	18.55	6.73	2.13	0.88	1.24	2.95	0.07	0.90	0.18	3.24	20.52	5.85	0.16	77.36	0.30	17.62
Sha-1	QL-10	PRM	63.15	15.84	6.54	2.85	1.04	3.03	3.15	0.10	0.42	0.10	3.99	37.71	2.52	0.20	63.23	0.32	9.71
Sha-1	QL-11	PRM	64.83	17.03	4.89	1.81	1.22	2.37	3.17	0.06	0.72	0.16	3.81	23.52	4.58	0.19	68.28	0.30	12.54
Sha-1	QL-12	PRM	62.41	17.20	5.92	1.91	0.98	1.26	4.22	0.06	0.71	0.12	3.63	24.12	4.96	0.25	71.85	0.35	14.53
Sha-2 average			63.75	15.43	5.40	1.80	2.31	1.82	3.34	0.08	0.69	0.16	3.99	22.75	5.12	0.21	68.76	0.28	12.81
Sha-1 average			60.18	17.85	6.68	2.23	1.10	1.30	3.38	0.07	0.77	0.16	3.40	23.72	4.66	0.19	74.89	0.33	16.24
Shaximiao Formation average			61.97	16.64	6.04	2.02	1.70	1.56	3.36	0.07	0.73	0.16	3.93	23.23	4.89	0.20	71.83	0.31	14.52
NASC			64.80	16.90	5.65	2.86	3.63	1.14	3.97	0.06	0.70	0.13	3.83	24.14	/	0.23	/	/	/
PAAS			62.80	18.90	7.22	2.20	1.30	1.20	3.70	0.11	1.00	0.16	3.32	18.90	/	0.20	/	/	/

### 4.3 Trace Element Geochemistry

**Figure 5:**
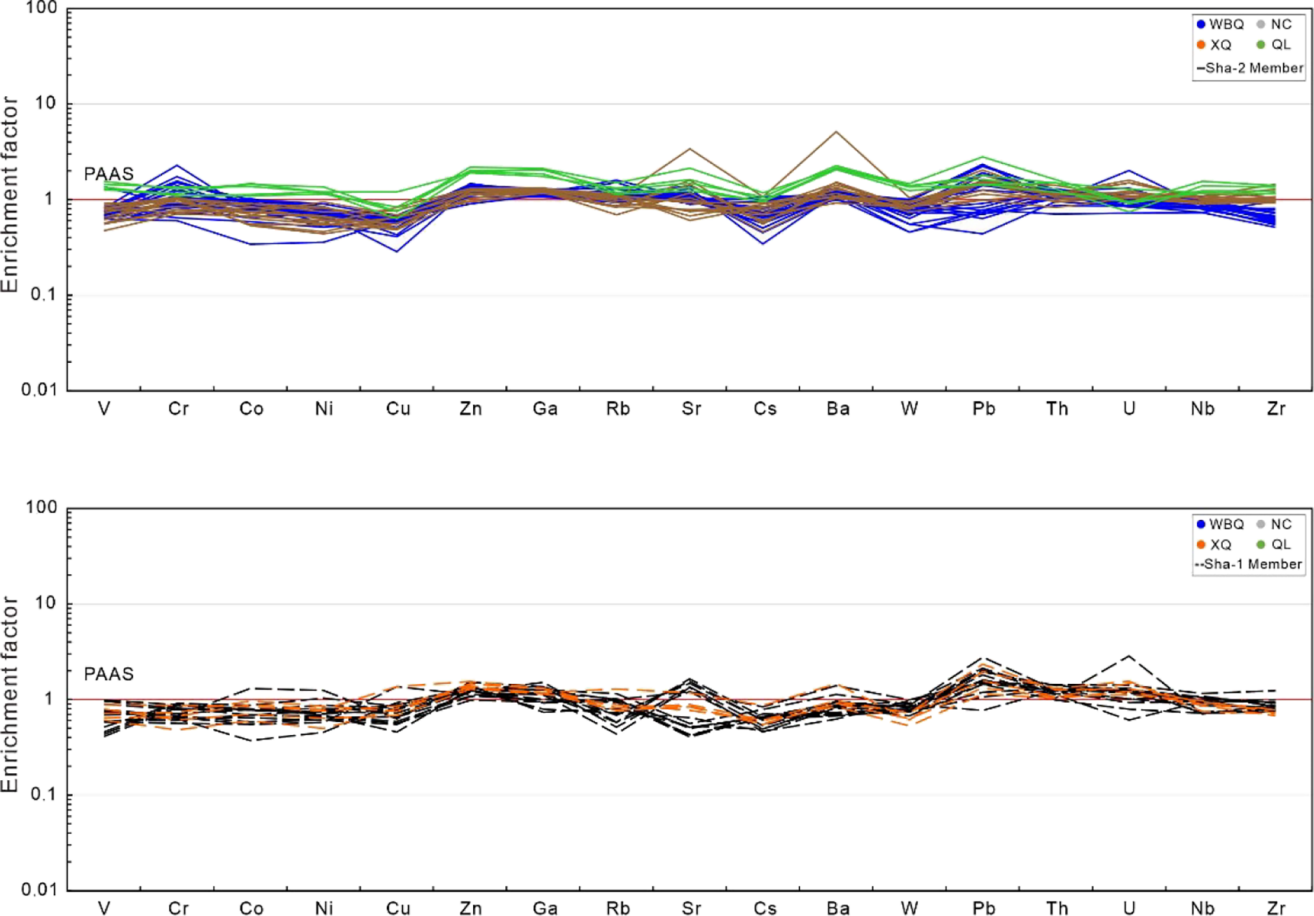
Enrichment factors of trace elements of mudstone samples from the Shaximiao Formation.

Table 2 shows the trace element data of mudstone samples from the Shaximiao Formation. Generally, the degree of enrichment or consumption of trace elements in the sample is evaluated relative to the concentration in the reference of the post-Archean Australian shale (PAAS).^25^ The average enrichment factors of Zn, Ga, Sr, and Pb are greater than 1, indicating relatively enriched. A few trace elements are characterized by enrichment factor less than 1 including V, Co, Ni, Cu, Cs, W, Nb, and Zr, all of which exhibit depleted concentrations. Other elements fall in the variable range of enrichment and depletion (Figure 5).

**Table 2:** Trace Elements Concentration (in ppm) of Mudstone Samples from the Shaximiao Formation

member	sample	mudstone litho.	V	Cr	Co	Ni	Cu	Zn	Ga	Rb	Sr	Cs	Ba	W	Pb
Sha-2	WBQ-1	PRM	80.40	55.50	11.20	24.30	31.60	76.30	16.30	109.00	226.00	10.10	796.00	1.44	179.00
Sha-2	WBQ-2	PRM	91.50	74.20	14.10	35.70	13.20	104.00	22.90	174.00	169.00	14.80	682.00	2.11	11.70
Sha-2	WBQ-3	PRM	105.00	65.20	12.40	25.90	27.30	98.40	22.50	149.00	181.00	11.40	765.00	1.37	8.06
Sha-2	WBQ-4	GGM	82.90	120.00	15.80	33.30	24.20	89.90	21.00	143.00	166.00	7.15	603.00	1.79	15.50
Sha-2	WBQ-5	PRM	86.30	131.00	15.60	35.60	21.90	103.00	21.90	177.00	188.00	9.37	683.00	2.26	37.80
Sha-2	WBQ-6	PRM	98.60	142.00	17.50	35.80	28.40	96.90	20.90	150.00	192.00	7.98	624.00	1.99	23.50
Sha-2	WBQ-7	PRM	94.10	141.00	17.80	31.00	27.10	95.70	20.00	149.00	174.00	7.13	626.00	1.63	12.40
Sha-2	WBQ-8	GGM	94.40	160.00	16.80	32.00	25.00	97.80	21.00	149.00	184.00	7.65	625.00	2.09	13.00
Sha-2	WBQ-9	PRM	89.70	116.00	16.80	34.10	24.30	95.50	21.00	163.00	181.00	9.29	595.00	1.97	32.90
Sha-2	WBQ-10	PRM	102.00	211.00	14.40	29.30	27.20	75.70	17.80	125.00	198.00	6.37	599.00	2.00	11.70
Sha-2	WBQ-11	GGM	99.20	91.20	16.60	36.50	27.40	93.10	21.50	171.00	168.00	11.40	678.00	2.38	42.40
Sha-2	WBQ-12	PRM	99.00	86.00	18.00	35.70	29.80	95.30	21.20	169.00	205.00	9.66	736.00	1.52	25.00
Sha-2	WBQ-13	PRM	108.00	92.20	15.80	30.80	26.70	78.40	20.40	133.00	197.00	6.26	614.00	1.24	12.30
Sha-2	WBQ-14	GGM	160.00	72.40	17.10	23.90	16.00	77.40	17.40	107.00	205.00	4.01	583.00	0.96	12.20
Sha-2	WBQ-15	GGM	97.30	84.80	20.30	33.10	24.50	99.10	19.90	133.00	195.00	5.78	574.00	1.05	12.10
Sha-2	WBQ-16	PRM	66.80	97.30	13.70	29.40	19.40	73.00	13.80	105.00	166.00	5.78	444.00	1.62	27.80
Sha-2	WBQ-17	GGM	74.70	52.70	6.27	15.70	24.50	61.10	17.80	192.00	142.00	11.50	628.00	2.06	20.10
Sha-2	WBQ-18	GGM	73.90	70.60	11.30	33.70	16.50	96.80	18.00	197.00	160.00	11.60	600.00	1.74	21.90
Sha-2	WBQ-19	PRM	65.60	73.70	14.20	22.40	23.10	79.80	17.00	163.00	150.00	9.61	680.00	1.59	13.20
Sha-2	WBQ-20	GGM	104.00	99.90	20.40	41.30	22.00	100.00	22.40	136.00	193.00	9.62	567.00	1.73	31.90
Sha-2	WBQ-21	GGM	108.00	107.00	20.60	41.90	28.40	113.00	22.80	150.00	195.00	9.38	737.00	1.72	29.80
Sha-2	WBQ-22	GGM	94.20	104.00	17.10	33.20	27.00	84.50	22.00	144.00	205.00	7.84	733.00	1.59	32.80
Sha-2	NC-1	PRM	109.00	87.60	19.70	38.60	36.30	101.00	23.90	128.00	149.00	11.70	917.00	2.09	23.70
Sha-2	NC-2	PRM	100.00	90.00	13.20	40.10	26.60	73.30	18.70	85.80	182.00	5.35	542.00	1.77	15.20
Sha-2	NC-3	PRM	113.00	85.00	13.30	25.60	34.20	92.10	21.90	135.00	559.00	13.20	2744.00	2.33	33.70
Sha-2	NC-4	PRM	95.40	89.20	10.10	20.40	26.40	65.70	19.00	110.00	230.00	6.97	704.00	1.60	16.00
Sha-2	NC-5	PRM	79.80	82.70	16.50	35.20	23.60	98.20	25.00	161.00	142.00	12.00	658.00	2.04	34.60
Sha-2	NC-6	PRM	77.50	93.70	16.50	28.60	23.30	80.50	21.30	132.00	163.00	8.97	777.00	1.93	25.80
Sha-2	NC-7	PRM	65.70	85.70	9.90	23.10	25.30	62.30	18.20	103.00	249.00	6.46	771.00	1.75	17.80
Sha-2	NC-8	PRM	66.30	64.50	13.40	28.40	22.00	77.70	19.50	140.00	148.00	10.90	660.00	1.97	24.00
Sha-2	NC-9	PRM	64.10	71.00	14.00	29.90	22.80	93.60	22.60	156.00	123.00	12.20	682.00	1.98	26.40
Sha-2	NC-10	PRM	76.40	87.30	11.40	23.90	18.90	68.00	19.10	106.00	163.00	7.15	525.00	1.71	23.00
Sha-2	NC-11	PRM	87.40	94.20	9.95	19.40	22.80	71.00	20.80	141.00	150.00	9.34	669.00	1.99	25.00
Sha-2	NC-12	PRM	101.00	70.00	16.30	38.50	28.20	91.70	22.20	127.00	132.00	8.93	513.00	1.92	26.60
Sha-2	NC-13	PRM	100.00	93.20	17.10	38.00	20.60	85.50	21.30	150.00	99.60	10.40	581.00	2.12	25.60
Sha-2	QL-1	GGM	133.76	79.21	17.08	43.54	24.48	121.87	27.80	124.06	174.40	9.32	924.38	2.46	23.51
Sha-2	QL-2	GGM	133.50	83.99	17.32	43.37	28.78	114.50	25.55	125.25	171.03	9.80	924.06	2.31	19.93
Sha-2	QL-3	PRM	143.13	87.15	20.46	45.92	23.15	105.20	25.02	125.30	182.73	9.87	904.75	2.32	20.07
Sha-2	QL-4	PRM	129.30	86.89	23.21	44.69	21.46	110.40	23.85	144.06	221.37	10.16	930.06	2.50	19.47
Sha-2	QL-5	PRM	136.45	93.36	19.91	41.86	37.86	101.99	26.66	150.35	268.56	11.15	871.17	2.50	35.24
Sha-1	XQ-1	GGM	81.50	47.20	12.30	24.50	36.00	101.00	21.20	184.00	210.00	11.60	846.00	1.48	35.00
Sha-1	XQ-2	DGM	106.00	79.40	21.90	46.30	36.90	123.00	25.50	121.00	166.00	8.10	588.00	1.96	31.10
Sha-1	XQ-3	DGM	126.00	71.20	17.10	36.80	35.90	115.00	24.20	149.00	163.00	9.56	579.00	1.51	24.20
Sha-1	XQ-4	DGM	126.00	91.30	18.40	39.60	40.60	112.00	22.30	118.00	162.00	7.94	557.00	1.62	25.10
Sha-1	XQ-5	DGM	99.70	75.90	21.10	44.70	70.30	136.00	27.20	136.00	183.00	9.43	651.00	2.02	48.30
Sha-1	NC-14	DGM	60.00	87.50	16.40	40.90	36.30	97.00	17.10	150.00	91.70	9.13	441.00	1.98	29.40
Sha-1	NC-15	DGM	90.20	67.20	16.00	36.70	29.50	91.10	23.40	163.00	83.50	10.60	446.00	2.07	28.80
Sha-1	NC-16	DGM	56.40	82.60	14.50	38.80	36.40	96.20	16.80	145.00	93.40	8.91	426.00	1.95	29.00
Sha-1	NC-17	DGM	64.00	83.50	16.70	47.30	35.10	98.40	12.20	107.00	87.50	6.12	472.00	1.94	46.00
Sha-1	NC-18	DGM	55.10	79.30	14.30	31.20	31.50	86.80	12.70	93.70	104.00	5.61	326.00	2.12	23.90
Sha-1	NC-19	DGM	85.00	60.10	12.30	35.30	27.30	81.00	18.40	146.00	78.60	9.53	431.00	2.00	23.10
Sha-1	NC-20	DGM	88.80	65.50	15.60	35.30	32.40	91.50	19.70	149.00	84.70	9.34	432.00	2.07	38.30
Sha-1	NC-21	DGM	84.90	67.80	14.20	31.60	32.30	100.00	19.20	150.00	97.40	9.48	524.00	2.19	28.90
Sha-1	NC-22	GGM	89.40	67.70	15.50	41.40	35.10	92.30	21.80	159.00	96.30	10.20	636.00	2.06	13.30
Sha-1	QL-6	DGM	143.14	93.99	17.85	36.54	22.10	105.84	24.41	154.90	233.42	12.16	879.17	2.59	30.08
Sha-1	QL-7	DGM	117.58	71.40	8.84	25.81	48.09	114.10	23.85	115.11	305.98	9.40	638.40	2.13	37.53
Sha-1	QL-8	DGM	118.13	75.94	13.31	33.46	71.90	100.97	24.33	97.39	256.56	7.83	572.74	2.12	22.45
Sha-1	QL-9	DGM	108.52	72.86	13.17	33.07	26.98	100.29	22.05	68.56	261.54	6.73	521.29	1.79	28.41
Sha-1	QL-10	PRM	121.26	74.49	15.09	34.02	37.07	87.61	25.34	77.87	275.89	8.30	504.33	2.08	35.56
Sha-1	QL-11	PRM	121.12	77.82	16.09	36.98	32.55	95.67	24.95	98.59	281.05	8.18	564.00	2.00	36.54
Sha-1	QL-12	PRM	106.93	68.13	27.33	62.53	28.64	117.52	24.93	74.84	246.96	7.26	516.50	1.92	36.44
Sha-2 average			97.18	94.31	15.58	32.59	25.06	89.98	21.05	140.80	188.69	9.19	736.74	1.88	26.57
Sha-1 average			97.60	74.32	16.09	37.75	37.28	102.06	21.50	126.57	169.64	8.83	550.07	1.98	31.02
Shaximiao Formation average			97.39	84.32	15.84	35.17	31.17	96.02	21.27	133.68	179.17	9.01	643.40	1.93	28.79
PAAS			150.00	110.00	23.00	55.00	50.00	85.00	20.00	160.00	200.00	15.00	650.00	2.70	20.00

member	sample	mudstone litho.	Th	U	Nb	Zr	Hf	Ni/Co	U//Th	V/Cr	V/(V + Ni)	Sr/Cu	Sr/Ba	Th/U	Rb/Sr	1000Rb/K_2_O
Sha-2	WBQ-1	PRM	10.20	5.01	12.20	111.00	3.19	2.17	0.49	1.45	0.77	7.15	0.28	2.04	0.48	3.56
Sha-2	WBQ-2	PRM	14.50	2.40	14.10	157.00	3.68	2.53	0.17	1.23	0.72	12.80	0.25	6.04	1.03	4.05
Sha-2	WBQ-3	PRM	12.80	2.45	12.80	186.00	4.23	2.09	0.19	1.61	0.80	6.63	0.24	5.22	0.82	4.07
Sha-2	WBQ-4	GGM	14.60	2.54	16.90	106.00	3.31	2.11	0.17	0.69	0.71	6.86	0.28	5.75	0.86	3.77
Sha-2	WBQ-5	PRM	15.70	2.41	14.80	107.00	3.34	2.28	0.15	0.66	0.71	8.58	0.28	6.51	0.94	4.44
Sha-2	WBQ-6	PRM	13.00	2.65	15.20	103.00	3.40	2.05	0.20	0.69	0.73	6.76	0.31	4.91	0.78	4.08
Sha-2	WBQ-7	PRM	12.50	2.43	15.40	101.00	3.08	1.74	0.19	0.67	0.75	6.42	0.28	5.14	0.86	3.89
Sha-2	WBQ-8	GGM	14.50	2.85	15.50	109.00	3.44	1.90	0.20	0.59	0.75	7.36	0.29	5.09	0.81	3.88
Sha-2	WBQ-9	PRM	14.00	2.61	15.20	112.00	3.77	2.03	0.19	0.77	0.72	7.45	0.30	5.36	0.90	4.13
Sha-2	WBQ-10	PRM	13.60	2.69	13.10	95.70	3.04	2.03	0.20	0.48	0.78	7.28	0.33	5.06	0.63	3.41
Sha-2	WBQ-11	GGM	16.50	3.77	13.80	111.00	3.26	2.20	0.23	1.09	0.73	6.13	0.25	4.38	1.02	3.85
Sha-2	WBQ-12	PRM	15.40	2.75	14.30	127.00	3.84	1.98	0.18	1.15	0.73	6.88	0.28	5.60	0.82	4.14
Sha-2	WBQ-13	PRM	13.10	2.64	14.30	111.00	3.26	1.95	0.20	1.17	0.78	7.38	0.32	4.96	0.68	3.79
Sha-2	WBQ-14	GGM	8.01	1.74	10.80	84.20	2.53	1.40	0.22	2.21	0.87	12.81	0.35	4.60	0.52	3.35
Sha-2	WBQ-15	GGM	10.80	2.26	13.60	99.40	2.68	1.63	0.21	1.15	0.75	7.96	0.34	4.78	0.68	3.54
Sha-2	WBQ-16	PRM	8.54	5.55	9.79	76.10	2.11	2.15	0.65	0.69	0.69	8.56	0.37	1.54	0.63	3.78
Sha-2	WBQ-17	GGM	13.00	2.11	13.70	130.00	3.79	2.50	0.16	1.42	0.83	5.80	0.23	6.16	1.35	4.28
Sha-2	WBQ-18	GGM	13.40	2.44	13.80	116.00	3.78	2.98	0.18	1.05	0.69	9.70	0.27	5.49	1.23	4.50
Sha-2	WBQ-19	PRM	15.00	2.44	14.80	122.00	3.75	1.58	0.16	0.89	0.75	6.49	0.22	6.15	1.09	4.00
Sha-2	WBQ-20	GGM	15.60	2.37	15.60	122.00	3.44	2.02	0.15	1.04	0.72	8.77	0.34	6.58	0.70	4.17
Sha-2	WBQ-21	GGM	15.10	3.01	17.20	113.00	3.32	2.03	0.20	1.01	0.72	6.87	0.26	5.02	0.77	3.76
Sha-2	WBQ-22	GGM	14.50	2.94	15.50	131.00	3.86	1.94	0.20	0.91	0.74	7.59	0.28	4.93	0.70	3.93
Sha-2	NC-1	PRM	13.60	3.62	16.80	202.00	6.11	1.96	0.27	1.24	0.74	4.10	0.16	3.76	0.86	4.13
Sha-2	NC-2	PRM	13.00	2.77	16.00	234.00	6.68	3.04	0.21	1.11	0.71	6.84	0.34	4.69	0.47	3.73
Sha-2	NC-3	PRM	13.30	3.83	15.80	181.00	5.48	1.92	0.29	1.33	0.82	16.35	0.20	3.47	0.24	4.26
Sha-2	NC-4	PRM	9.71	2.59	13.70	162.00	4.73	2.02	0.27	1.07	0.82	8.71	0.33	3.75	0.48	3.81
Sha-2	NC-5	PRM	14.60	3.11	17.20	214.00	6.34	2.13	0.21	0.96	0.69	6.02	0.22	4.69	1.13	3.99
Sha-2	NC-6	PRM	11.50	2.78	14.90	173.00	5.60	1.73	0.24	0.83	0.73	7.00	0.21	4.14	0.81	3.56
Sha-2	NC-7	PRM	11.20	2.65	14.10	175.00	5.41	2.33	0.24	0.77	0.74	9.84	0.32	4.23	0.41	3.53
Sha-2	NC-8	PRM	13.50	3.92	15.00	160.00	4.75	2.12	0.29	1.03	0.70	6.73	0.22	3.44	0.95	3.95
Sha-2	NC-9	PRM	14.70	3.34	15.80	176.00	5.34	2.14	0.23	0.90	0.68	5.39	0.18	4.40	1.27	3.78
Sha-2	NC-10	PRM	13.10	2.84	15.20	193.00	5.82	2.10	0.22	0.88	0.76	8.62	0.31	4.61	0.65	4.16
Sha-2	NC-11	PRM	13.90	3.73	16.50	218.00	6.15	1.95	0.27	0.93	0.82	6.58	0.22	3.73	0.94	4.22
Sha-2	NC-12	PRM	14.30	2.80	17.80	178.00	5.25	2.36	0.20	1.44	0.72	4.68	0.26	5.11	0.96	4.52
Sha-2	NC-13	PRM	16.90	2.68	19.00	180.00	5.57	2.22	0.16	1.07	0.72	4.83	0.17	6.31	1.51	4.62
Sha-2	QL-1	GGM	14.23	2.62	14.78	158.42	4.99	2.55	0.18	1.69	0.75	7.12	0.19	5.44	0.71	5.63
Sha-2	QL-2	GGM	12.06	2.04	15.29	167.12	5.20	2.50	0.17	1.59	0.75	5.94	0.19	5.90	0.73	4.92
Sha-2	QL-3	PRM	11.56	1.42	16.31	175.58	5.29	2.24	0.12	1.64	0.76	7.89	0.20	8.16	0.69	5.36
Sha-2	QL-4	PRM	14.93	1.92	16.06	176.67	5.31	1.93	0.13	1.49	0.74	10.32	0.24	7.78	0.65	4.33
Sha-2	QL-5	PRM	14.85	1.83	18.63	185.98	5.74	2.10	0.12	1.46	0.77	7.09	0.31	8.14	0.56	4.91
Sha-1	XQ-1	GGM	17.00	4.31	12.70	133.00	3.77	1.99	0.25	1.73	0.77	5.83	0.25	3.94	0.88	4.39
Sha-1	XQ-2	DGM	15.60	3.43	18.20	149.00	4.50	2.11	0.22	1.34	0.70	4.50	0.28	4.55	0.73	4.14
Sha-1	XQ-3	DGM	15.90	4.00	17.40	150.00	4.08	2.15	0.25	1.77	0.77	4.54	0.28	3.98	0.91	4.42
Sha-1	XQ-4	DGM	14.80	4.34	16.00	155.00	4.43	2.15	0.29	1.38	0.76	3.99	0.29	3.41	0.73	3.96
Sha-1	XQ-5	DGM	19.00	4.15	18.50	170.00	4.84	2.12	0.22	1.31	0.69	2.60	0.28	4.58	0.74	4.29
Sha-1	NC-14	DGM	16.70	3.46	18.30	179.00	5.28	2.49	0.21	0.69	0.59	2.53	0.21	4.83	1.64	4.20
Sha-1	NC-15	DGM	18.70	3.62	18.40	161.00	5.02	2.29	0.19	1.34	0.71	2.83	0.19	5.17	1.95	4.11
Sha-1	NC-16	DGM	16.70	3.37	18.20	176.00	5.28	2.68	0.20	0.68	0.59	2.57	0.22	4.96	1.55	4.15
Sha-1	NC-17	DGM	15.20	3.18	15.60	173.00	5.35	2.83	0.21	0.77	0.58	2.49	0.19	4.78	1.22	3.68
Sha-1	NC-18	DGM	16.80	3.51	17.70	209.00	6.52	2.18	0.21	0.69	0.64	3.30	0.32	4.79	0.90	3.82
Sha-1	NC-19	DGM	18.50	3.80	17.30	155.00	5.10	2.87	0.21	1.41	0.71	2.88	0.18	4.87	1.86	3.91
Sha-1	NC-20	DGM	20.30	3.71	17.30	166.00	5.56	2.26	0.18	1.36	0.72	2.61	0.20	5.47	1.76	3.92
Sha-1	NC-21	DGM	19.80	3.75	19.30	172.00	5.66	2.23	0.19	1.25	0.73	3.02	0.19	5.28	1.54	3.89
Sha-1	NC-22	GGM	15.90	2.70	17.80	154.00	5.14	2.67	0.17	1.32	0.68	2.74	0.15	5.89	1.65	4.33
Sha-1	QL-6	DGM	15.88	1.83	17.54	192.26	5.52	2.05	0.12	1.52	0.80	10.56	0.27	8.68	0.66	4.22
Sha-1	QL-7	DGM	15.70	9.14	13.89	193.22	5.81	2.92	0.58	1.65	0.82	6.36	0.48	1.72	0.38	3.67
Sha-1	QL-8	DGM	19.57	4.33	14.43	177.41	5.33	2.51	0.22	1.56	0.78	3.57	0.45	4.52	0.38	2.89
Sha-1	QL-9	DGM	14.17	2.41	13.41	154.49	4.41	2.51	0.17	1.49	0.77	9.69	0.50	5.89	0.26	2.32
Sha-1	QL-10	PRM	14.83	2.42	15.19	166.59	5.15	2.25	0.16	1.63	0.78	7.44	0.55	6.13	0.28	2.47
Sha-1	QL-11	PRM	15.15	2.84	14.76	157.93	4.83	2.30	0.19	1.56	0.77	8.63	0.50	5.34	0.35	3.11
Sha-1	QL-12	PRM	17.03	3.33	13.00	143.84	4.57	2.29	0.20	1.57	0.63	8.62	0.48	5.11	0.30	1.77
Sha-2 average			13.38	2.81	15.03	146.00	4.35	2.12	0.22	1.10	0.75	7.66	0.27	5.08	0.81	4.09
Sha-1 average			16.82	3.70	16.42	166.08	5.05	2.37	0.22	1.33	0.71	4.82	0.31	4.95	0.98	3.70
Shaximiao Formation average			15.10	3.26	15.73	156.04	4.70	2.25	0.22	1.22	0.73	6.24	0.29	5.01	0.90	3.90
PAAS			14.60	3.10	19.00	210.00	5.00									

Other indirect geochemical parameters are shown in Table 2 including Ni/Co, U/Th, V/Cr, Sr/Cu, Sr/Ba, V/(V + Ni), etc., most of which shows a co-evolution of trace element concentration and sedimentary environment. The Sr/Cu ratio is higher in the Sha-2 Member than in the Sha-1 Member, with average values of 7.66 and 6.82, respectively. Ni/Co, V/Cr, and Rb/Sr are slightly larger in Sha-1 Member, whereas Sr/Ba, U/Th, and V/(V + Ni) ratios are similar in the Sha-1 and Sha-2 Member.

### 4.4 Rare Earth Element Geochemistry

**Figure 6:**
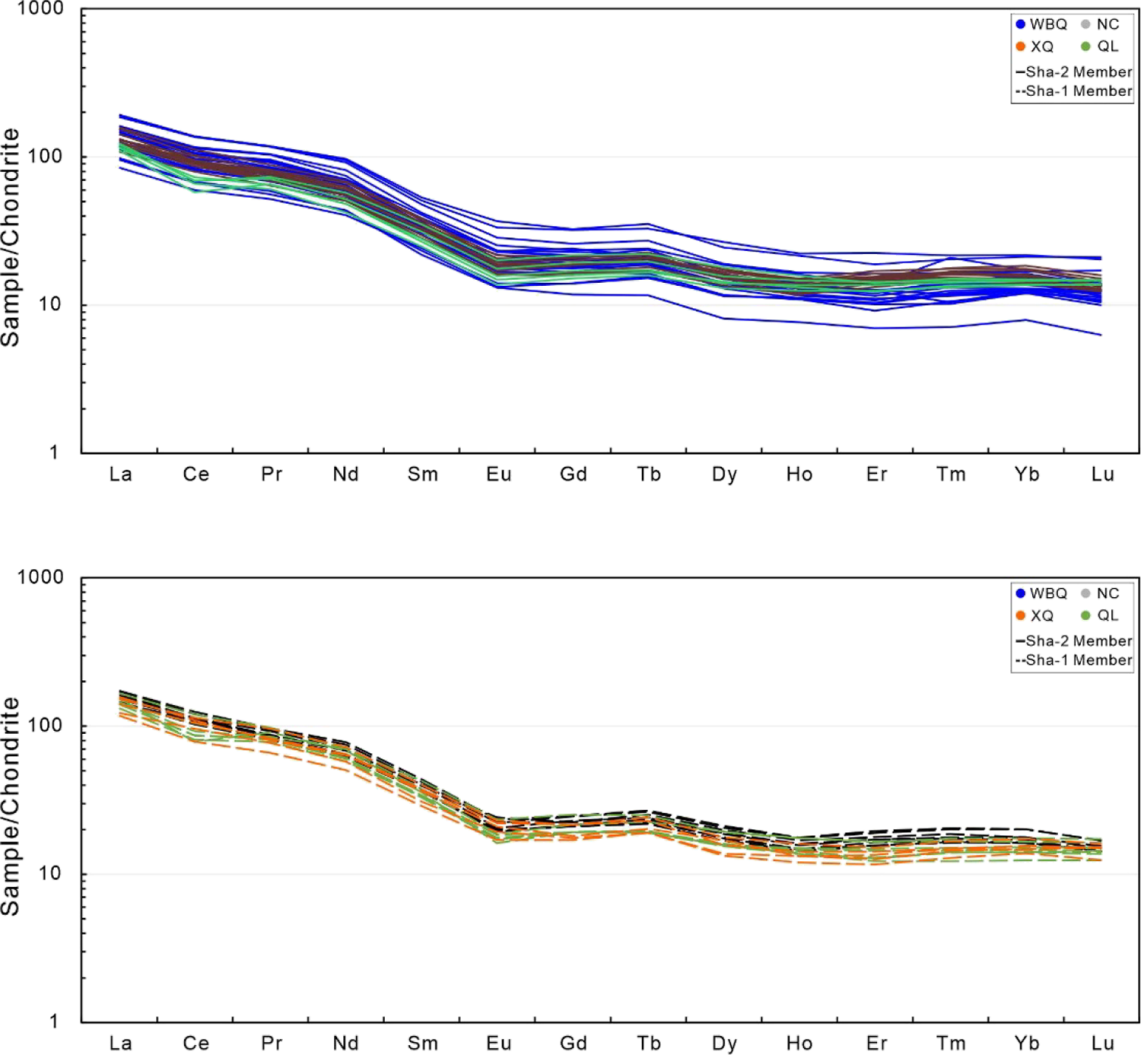
Chondrite-normalized REE patterns of mudstone from the Shaximiao Formation.^25^

The results of the REE concentration of the mudstone from the Shaximiao Formation are shown in Table 3. The chondrite-normalized REE patterns of the studied sample are given in Figure 6. The content of total rare earth elements (ΣREE) in mudstone from the Shaximiao Formation ranges from 125.35 to 286.49 ppm, with an average of 200.19 ppm, which is significantly higher than that of the upper continental crust (UCC; 146.37 ppm) and post-Archean Australian shale (PAAS; 184.77 ppm) (Table 3). The value of light rare earth element (LREE) ranges from 110.82 to 256.11 ppm, with an average value of 179.66 ppm, which is higher than the average value of North American Shale Composite (NASC) (152.84 ppm) and PAAS (167.36 ppm). The value of heavy rare earth element (HREE) ranges from 10.75 to 31.83 ppm, with an average of 20.53 ppm, lower than NASC (20.77 ppm), and higher than PAAS (17.6 ppm). The value range of ΣLREE/ ΣHREE is 7.63 to 10.36 ppm, with an average value of 8.78. The REE patterns of the chondrite normalized values in the Shaximiao Formation are marked by LREE enrichment, slightly negative Eu anomalies, and a rather flat HREE pattern (Figure 6). The characteristics of REE in Sha-1 and Sha-2 Member show no significant difference.

**Table 3:** Concentrations of Rare Earth Elements of Mudstone Samples from the Shaximiao Formation

member	sample	mudstone litho.	La	Ce	Pr	Nd	Sm	Eu	Gd	Tb	Dy	Ho	Er	Tm	Yb	Lu	ΣREE	ΣLREE	ΣHREE	ΣLREE/ΣHREE
Sha-2	WBQ-1	PRM	26.20	48.20	6.35	24.30	4.74	1.03	4.12	0.71	3.77	0.79	1.92	0.34	2.51	0.37	125.35	110.82	14.53	7.63
Sha-2	WBQ-2	PRM	50.00	94.30	12.70	48.90	8.19	1.86	6.93	1.13	6.00	1.16	2.87	0.68	3.60	0.45	238.77	215.95	22.82	9.46
Sha-2	WBQ-3	PRM	34.80	65.80	8.34	30.80	5.81	1.29	5.22	0.95	4.82	0.97	2.65	0.44	3.39	0.45	165.73	146.84	18.89	7.77
Sha-2	WBQ-4	GGM	30.40	54.90	7.16	25.50	4.56	0.98	4.13	0.74	3.70	0.81	2.15	0.38	2.61	0.34	138.35	123.50	14.85	8.31
Sha-2	WBQ-5	PRM	36.80	72.80	9.24	34.30	6.65	1.47	5.90	0.95	4.89	1.02	2.54	0.45	3.40	0.43	180.85	161.26	19.59	8.23
Sha-2	WBQ-6	PRM	45.70	82.50	10.10	38.90	6.35	1.61	5.59	0.92	4.88	0.99	2.60	0.45	2.99	0.44	204.02	185.16	18.86	9.82
Sha-2	WBQ-7	PRM	40.70	74.10	10.10	35.20	6.47	1.32	4.91	0.90	4.52	0.83	2.26	0.41	2.67	0.36	184.75	167.89	16.86	9.96
Sha-2	WBQ-8	GGM	47.30	89.00	11.20	40.60	6.96	1.44	5.95	1.00	5.24	0.96	2.88	0.48	3.22	0.44	216.67	196.50	20.17	9.74
Sha-2	WBQ-9	PRM	38.50	77.90	9.01	35.60	6.00	1.47	5.36	0.91	4.56	0.88	2.64	0.38	2.66	0.38	186.25	168.48	17.77	9.48
Sha-2	WBQ-10	PRM	44.20	85.20	10.40	40.80	6.71	1.43	6.09	0.92	5.01	0.93	2.42	0.43	2.71	0.40	207.64	188.74	18.90	9.99
Sha-2	WBQ-11	GGM	50.00	92.10	12.60	44.80	7.25	1.70	6.39	0.98	5.15	0.92	2.51	0.45	2.85	0.41	228.12	208.45	19.67	10.60
Sha-2	WBQ-12	PRM	59.50	111.00	14.40	56.90	9.91	2.45	9.48	1.54	8.58	1.60	4.72	0.70	4.55	0.66	285.99	254.16	31.83	7.99
Sha-2	WBQ-13	PRM	45.50	78.60	10.30	40.70	7.56	1.70	7.10	1.01	4.88	0.89	2.59	0.34	2.63	0.34	204.14	184.36	19.78	9.32
Sha-2	WBQ-14	GGM	29.70	54.10	6.81	26.20	4.28	0.97	3.48	0.55	2.61	0.55	1.47	0.23	1.66	0.20	132.80	122.06	10.75	11.36
Sha-2	WBQ-15	GGM	57.50	110.00	14.30	55.10	9.35	2.10	7.66	1.28	6.10	1.19	3.42	0.53	3.51	0.55	272.60	248.35	24.25	10.24
Sha-2	WBQ-16	PRM	39.30	68.90	9.29	32.70	5.83	1.24	4.86	0.77	3.77	0.78	2.12	0.33	2.52	0.32	172.73	157.26	15.47	10.16
Sha-2	WBQ-17	GGM	47.10	86.10	11.50	41.10	6.96	1.47	5.95	0.96	4.86	0.88	2.56	0.47	2.94	0.37	213.21	194.23	18.98	10.23
Sha-2	WBQ-18	GGM	59.30	111.00	14.40	58.30	10.40	2.71	9.60	1.66	7.87	1.55	3.94	0.66	4.42	0.68	286.49	256.11	30.38	8.43
Sha-2	WBQ-19	PRM	43.90	84.70	11.20	42.50	7.94	1.72	6.76	1.04	5.03	0.93	2.65	0.43	2.89	0.44	212.13	191.96	20.17	9.52
Sha-2	WBQ-20	GGM	36.60	67.20	8.33	33.10	6.09	1.36	5.20	0.88	4.27	0.85	2.32	0.37	2.54	0.35	169.46	152.68	16.78	9.10
Sha-2	WBQ-21	GGM	36.40	72.90	9.56	34.40	6.34	1.47	5.57	0.90	4.16	0.80	2.18	0.39	2.62	0.40	178.09	161.07	17.02	9.47
Sha-2	WBQ-22	GGM	48.30	83.80	11.70	42.00	7.39	1.68	6.28	1.11	5.15	1.00	2.88	0.46	3.04	0.45	215.24	194.87	20.37	9.57
Sha-2	NC-1	PRM	38.30	73.90	8.70	33.00	6.32	1.43	5.57	0.99	5.39	1.03	3.15	0.55	3.60	0.46	182.39	161.65	20.74	7.79
Sha-2	NC-2	PRM	37.20	70.00	8.41	31.60	5.77	1.17	4.98	0.85	4.58	0.86	2.93	0.48	3.01	0.41	172.25	154.15	18.10	8.52
Sha-2	NC-3	PRM	38.50	72.20	8.53	31.30	5.51	1.20	4.85	0.81	4.58	0.94	3.27	0.58	3.85	0.51	176.62	157.24	19.38	8.11
Sha-2	NC-4	PRM	40.40	81.50	10.00	40.10	7.32	1.59	6.22	1.06	5.57	1.02	3.22	0.50	3.05	0.40	201.95	180.91	21.04	8.60
Sha-2	NC-5	PRM	48.00	82.70	9.83	37.90	7.04	1.52	6.12	1.05	5.58	1.08	3.57	0.57	3.67	0.49	209.12	186.99	22.13	8.45
Sha-2	NC-6	PRM	38.20	75.40	9.13	35.90	7.04	1.48	5.87	1.02	5.23	0.98	3.20	0.52	3.35	0.43	187.75	167.15	20.60	8.11
Sha-2	NC-7	PRM	33.50	64.20	7.81	30.30	5.47	1.17	4.68	0.81	4.37	0.84	2.78	0.48	2.94	0.40	159.74	142.45	17.29	8.24
Sha-2	NC-8	PRM	38.90	74.40	8.75	33.70	6.34	1.30	5.52	0.92	4.93	0.98	3.13	0.53	3.26	0.44	183.10	163.39	19.71	8.29
Sha-2	NC-9	PRM	38.80	72.10	8.63	33.30	6.27	1.26	5.45	0.94	4.84	0.96	3.05	0.50	3.14	0.42	179.66	160.36	19.30	8.31
Sha-2	NC-10	PRM	40.50	76.50	9.46	36.20	6.73	1.35	5.66	0.94	4.87	0.93	2.97	0.47	2.94	0.40	189.92	170.74	19.18	8.90
Sha-2	NC-11	PRM	41.00	75.00	9.14	33.50	6.42	1.38	5.58	0.97	5.15	0.99	3.09	0.52	3.25	0.44	186.44	166.44	20.00	8.32
Sha-2	NC-12	PRM	44.30	76.80	9.71	37.30	6.91	1.49	6.22	1.05	5.59	1.05	3.42	0.52	3.35	0.44	198.15	176.51	21.64	8.16
Sha-2	NC-13	PRM	48.90	90.60	10.80	41.10	7.51	1.48	6.29	1.06	5.61	1.06	3.26	0.53	3.38	0.43	222.01	200.39	21.62	9.27
Sha-2	QL-1	GGM	38.36	55.64	8.98	34.47	6.88	1.48	6.42	1.04	5.81	1.14	2.96	0.49	3.13	0.48	167.28	145.82	21.46	6.80
Sha-2	QL-2	GGM	34.32	46.72	8.00	29.05	5.25	1.09	4.72	0.79	4.65	0.95	2.57	0.44	2.88	0.44	141.87	124.44	17.43	7.14
Sha-2	QL-3	PRM	37.60	56.29	8.70	31.14	6.13	1.29	5.70	0.93	5.03	1.06	3.01	0.47	3.07	0.47	160.90	141.16	19.74	7.15
Sha-2	QL-4	PRM	36.10	59.16	7.91	28.94	5.49	1.15	5.03	0.81	4.54	0.98	2.87	0.46	3.04	0.47	156.96	138.76	18.20	7.62
Sha-2	QL-5	PRM	35.71	53.43	7.42	25.43	4.86	1.01	4.48	0.75	4.20	0.90	2.64	0.43	2.85	0.45	144.55	127.86	16.69	7.66
Sha-1	XQ-1	GGM	36.50	63.60	8.05	30.40	5.65	1.25	5.02	0.90	4.31	0.86	2.45	0.42	2.90	0.40	162.71	145.45	17.26	8.43
Sha-1	XQ-2	DGM	43.60	85.30	10.10	38.90	7.32	1.63	6.38	1.08	5.08	1.02	2.99	0.48	3.25	0.49	207.62	186.85	20.77	8.99
Sha-1	XQ-3	DGM	49.00	88.70	9.82	38.10	7.12	1.54	5.36	0.95	5.49	0.95	2.84	0.48	3.08	0.50	213.93	194.28	19.65	9.89
Sha-1	XQ-4	DGM	38.00	77.60	9.41	34.60	6.05	1.42	5.20	0.90	4.44	0.96	2.65	0.47	3.11	0.48	185.29	167.08	18.21	9.18
Sha-1	XQ-5	DGM	47.30	91.90	11.70	43.30	7.85	1.69	6.38	1.14	5.73	1.15	3.19	0.54	3.63	0.52	226.02	203.74	22.28	9.14
Sha-1	NC-14	DGM	45.40	83.30	9.87	37.60	7.11	1.44	6.22	1.03	5.43	1.04	3.30	0.53	3.45	0.46	206.18	184.72	21.46	8.61
Sha-1	NC-15	DGM	47.20	89.20	10.40	38.90	7.19	1.45	6.24	1.06	5.74	1.07	3.44	0.55	3.42	0.46	216.32	194.34	21.98	8.84
Sha-1	NC-16	DGM	44.90	83.10	10.10	38.40	7.16	1.48	6.22	1.04	5.45	1.03	3.26	0.54	3.40	0.46	206.54	185.14	21.40	8.65
Sha-1	NC-17	DGM	48.80	90.90	10.60	41.50	7.34	1.43	6.34	1.10	5.62	1.06	3.29	0.54	3.42	0.46	222.40	200.57	21.83	9.19
Sha-1	NC-18	DGM	50.00	92.10	11.30	42.80	7.88	1.51	6.80	1.13	6.02	1.13	3.61	0.58	3.71	0.50	229.06	205.59	23.47	8.76
Sha-1	NC-19	DGM	44.50	87.70	10.50	41.10	7.91	1.65	6.66	1.19	6.41	1.22	3.75	0.61	3.71	0.48	217.39	193.36	24.03	8.05
Sha-1	NC-20	DGM	53.70	101.00	11.80	46.90	8.59	1.72	7.30	1.27	6.84	1.27	4.12	0.66	4.22	0.54	249.94	223.71	26.23	8.53
Sha-1	NC-21	DGM	53.50	97.20	11.60	45.10	8.22	1.64	7.24	1.24	6.63	1.26	4.00	0.65	4.21	0.54	243.04	217.26	25.78	8.43
Sha-1	NC-22	GGM	51.80	91.00	10.70	41.20	7.71	1.78	6.62	1.14	6.09	1.16	3.62	0.57	3.59	0.46	227.43	204.19	23.24	8.78
Sha-1	QL-6	DGM	45.04	74.45	10.34	41.94	8.35	1.72	7.44	1.17	6.16	1.26	3.50	0.54	3.47	0.52	205.88	181.83	24.05	7.56
Sha-1	QL-7	DGM	47.77	63.12	11.04	40.88	6.96	1.19	5.66	0.90	5.04	0.99	2.68	0.45	2.96	0.45	190.10	170.97	19.13	8.94
Sha-1	QL-8	DGM	51.44	96.66	11.91	42.05	7.34	1.38	6.36	1.06	6.25	1.26	3.43	0.57	3.64	0.56	233.90	210.77	23.13	9.11
Sha-1	QL-9	DGM	43.52	69.46	10.15	36.38	6.66	1.35	5.68	0.90	4.99	0.98	2.57	0.40	2.59	0.40	186.02	167.51	18.51	9.05
Sha-1	QL-10	PRM	43.41	70.01	9.87	36.02	6.43	1.26	5.65	0.92	5.07	1.08	3.11	0.49	3.17	0.49	186.98	167.00	19.98	8.36
Sha-1	QL-11	PRM	44.05	84.25	10.11	36.68	6.52	1.28	5.69	0.92	5.03	1.06	3.02	0.47	3.05	0.46	202.58	182.89	19.69	9.29
Sha-1	QL-12	PRM	40.82	65.42	9.53	34.73	6.51	1.30	5.67	0.91	5.17	1.03	2.71	0.46	2.92	0.44	177.62	158.31	19.31	8.20
Sha-2 average			41.41	75.54	9.72	36.67	6.63	1.46	5.25	1.52	5.01	0.97	2.83	0.47	3.09	0.43	191.00	171.43	19.57	8.80
Sha-1 average			46.20	83.14	10.42	39.40	7.23	1.48	4.51	2.73	5.57	1.09	3.22	0.52	3.38	0.48	209.38	187.88	21.49	8.76
Shaximiao Formation average			43.80	79.34	10.07	38.04	6.93	1.47	4.88	2.12	5.29	1.03	3.03	0.50	3.23	0.45	200.19	179.66	20.53	8.78
NASC			32.00	73.00	7.90	33.00	5.70	1.24	5.20	0.85	6.20	1.04	3.40	0.50	3.10	0.48	173.61	152.84	20.77	7.36
PAAS			38.00	80.00	8.83	33.90	5.55	1.08	4.66	0.77	4.68	0.99	2.85	0.40	2.82	0.43	184.96	167.36	17.60	9.51

## 5 Discussion

### 5.1 Provenance

**Figure 7:**
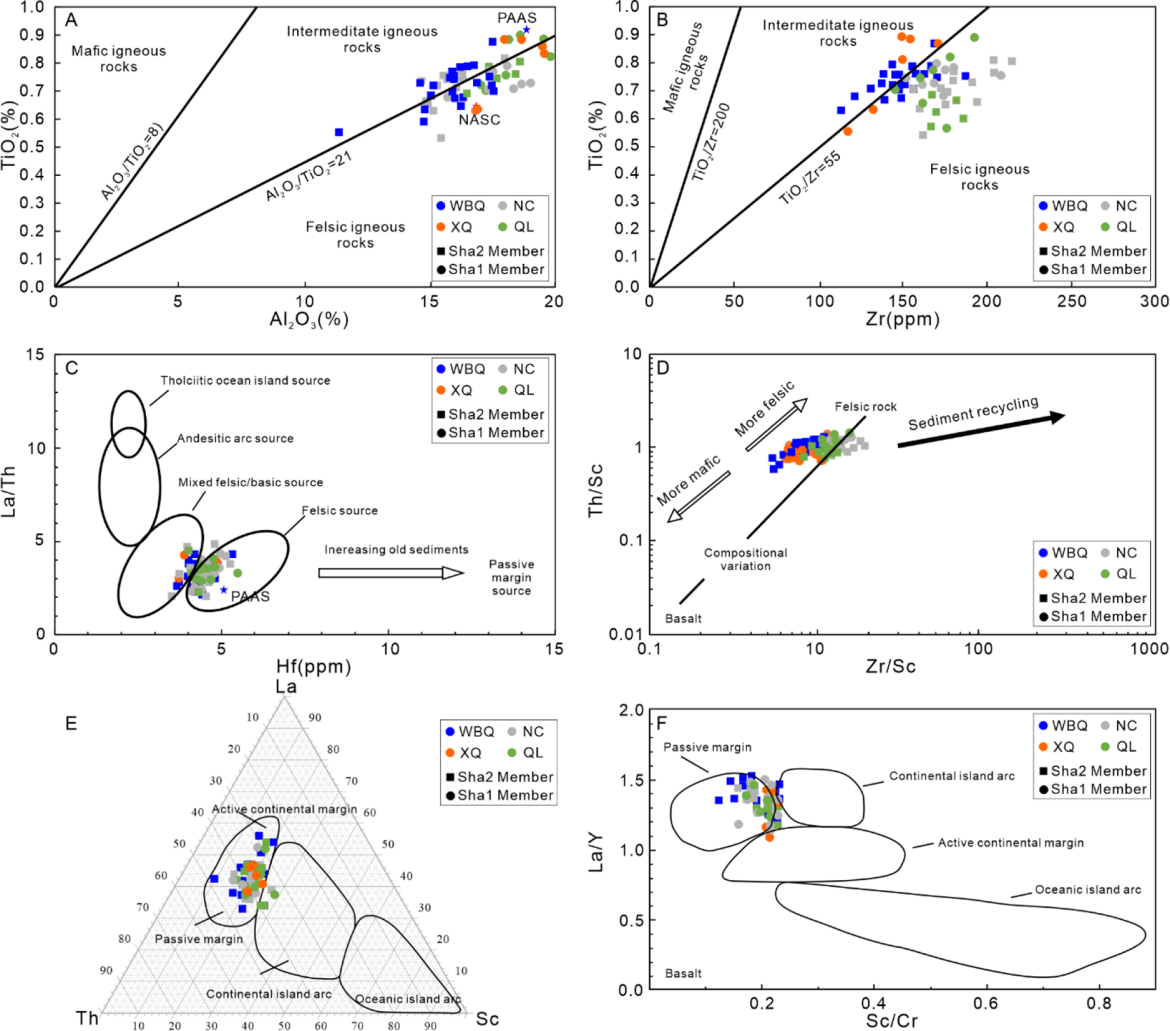
Provenance analysis and tectonic discrimination of the Shaximiao Formation mudstones. (A) TiO_2_ versus Al_2_O_3_ diagram;^41^ (B) TiO_2_ versus Zr binary plots; (C) La/Th versus Hf bivariate plot;^42^ (D) Th/Sc versus Zr/Sc bivariate plot;^43^ (E) La–Th–Sc ternary diagram;^46^ (F) Sc/Cr versus La/Y binary blot.^46^

The geochemistry of fine-grained clastic sediments has been widely used to identify the provenance of the sedimentary system in the sink area. The ratios of immobile elements such as Al^3+^, Zr^+^, and Ti^4+^ oxides in sedimentary rocks are usually similar to those of parental rocks.^31,38−40^ The high ratios of Al_2_O_3_/TiO_2_ for the studied samples range from 19.99 to 37.71 (average = 23.23), indicating sediments mainly derive from felsic parent rocks in the source area (Figure 7A).^41^ In addition, the TiO_2_/Zr ratio of the samples varies from 2.14 to 7.72 (average = 4.89), suggesting a dominant felsic and intermediate source composition (Figure 7B).^41^ A scatter plot of La/Th versus Hf suggests derivation from a source area composed of mixed felsic/basic and felsic sources (Figure 7C).^42^ McLennan et al. (1993) insisted that Zr/Sc and Th/Sc ratios can be used to understand the variations of sediments from the source region in mineral composition, degree of sorting, and heavy mineral content.^43^ In the mudstone samples, these values range from 6.42 to 11.26 (average = 8.09) and from 0.75 to 1.39 (average = 0.96), respectively. A bivariate plot of Zr/Sc versus Th/Sc suggests that the studied mudstone samples derive principally from felsic igneous rocks, and the data fall far from the sedimentary recycling line indicating the clastic materials have not experienced the process of sediment recycling (Figure 7D). Felsic source rocks generally respond to higher LREE/HREE ratios and negative Eu anomalies, whereas lower LREE/HREE ratios and the absence of Eu anomalies are characteristic of mafic rocks.^44^ The studied mudstone samples are extremely enriched in LREE (LREE/HREE = 9.40 on average) and deficient in HREE, with obviously slight negative anomalies (Eu anomaly = 1.51 on average), exhibiting a felsic provenance. These results uniformly indicate the studied mudstones of the Shaximiao Formation mainly derive from felsic and intermediate igneous rocks other than sediment recycling.

Trace elements whose concentration depends on their relative insensitivity to weathering and transportation processes provide important evidence to explain the tectonic environment.^25^ On the discrimination diagrams of La-Th-Sc,^45^ most of the data fall in or adjacent to the passive margin field (Figure 7E). The Sc/Cr versus La/Y binary plot shows that the majority of samples were plotted in the passive marginal setting with a few data been grouped into continental island arc, an active continental margin or mixed continental island arc-active continental margin (Figure 7F).^46^ In summary, the overall geochemical characteristics of the discriminate diagrams from the Shaximiao Formation suggest that the tectonic setting of the source area of the main provenance was most likely a passive margin. Such interpretations are supported by the widely accepted tectonic evolutionary model, which insists that the Sichuan Basin has evolved from a passive continental margin to a foreland basin since the Middle Triassic, affected by multiphase collisions of tectonic blocks.^21^ During this period, due to the influence of the Yanshan movement, the Qinling orogenic belt was napped and uplifted, and the felsic igneous material of the Micangshan-Dabashan, characterized by low quartz, high feldspar and high content of metamorphic rock debris, was denuded and transported, feeding sediments to the Sichuan Basin as a main provenance. At the same time, western provenance with high quartz, low feldspar, and relatively low metamorphic rock debris from Longmenshan also provided clastic materials to the basin.

### 5.2 Paleoclimate and Paleo-weathering Conditions

The paleoclimate determines the mineralogy and chemical composition of siliciclastic sediments and is the key factor controlling the weathering degree of sediments.^47,48^ The concentrations of Fe, V, Ni, Ba, Co, and other elements are very sensitive to paleoclimatic conditions. The changes in their contents and corresponding ratios can be used to analyze the changes in paleoclimate and judge the sedimentary environment of a specific period. Geochemical indicators such as CIA, major oxide ratio (Al_2_O_3_/SiO_2_), and climate-sensitive trace elements (e.g., Ga, Rb, Sr, and Cu) have been widely used to reconstruct paleoclimatic conditions.^2,49−51^

**Figure 8:**
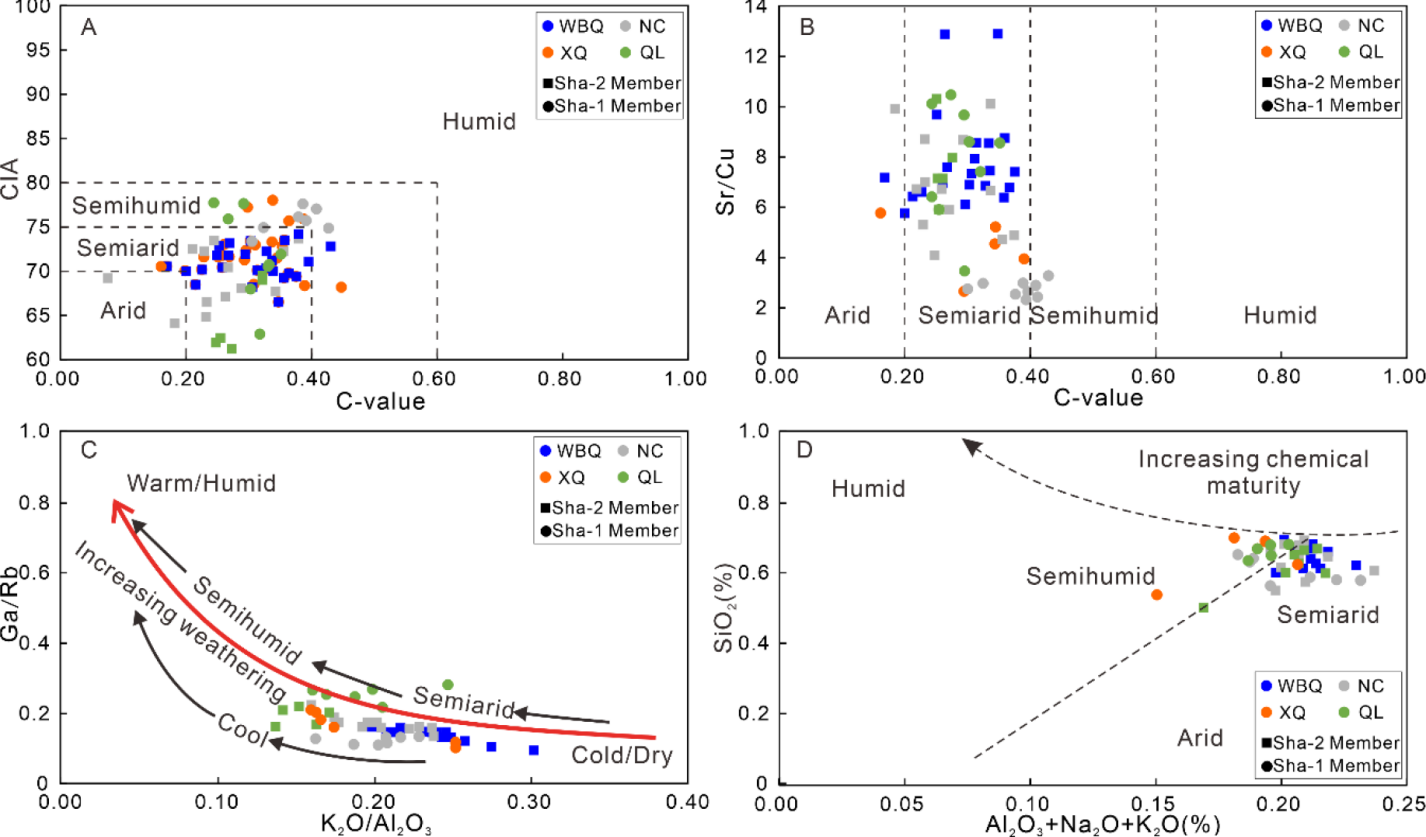
Analysis of paleoclimate conditions for the Shaximiao Formation mudstones. (A) C-value versus CIA diagram; (B) C-value versus Sr/Cu diagram; (C) K_2_O/Al_2_O_3_ versus Ga/Rb plot diagram;^52^ (D) Al_2_O_3_ + Na_2_O + K_2_O versus SiO_2_ discrimination diagrams.^81^

In this study, the CIA value of Sha-1 Member mudstone ranges from 63.23 to 77.88 (average = 74.89), reflecting moderate chemical weathering, belonging to a semi-humid climate. Differently, the CIA value of Sha-2 Member mudstone varies from 56.01 to 73.42 (average = 68.76), indicating a lower degree of chemical weathering than Sha-1 Member, which belongs to a semiarid and semihumid climate (Figure 8A). The ratio of wet elements (e.g., Rb and Cu) to dry elements(e.g., Sr)is often used to analyze the paleoclimatic characteristics. The high Sr content of sedimentary rocks may be caused by the concentration and precipitation of lake water under dry and hot climate conditions.^11,52^ A moderate Sr/Cu ratio (4.10–12.81, average = 7.66) and low C-value (0.07–0.45, average = 0.28) in Sha-2 Member support the interpretation of semiarid paleoenvironment, whereas a low Sr/Cu ratio (2.53–10.56, average = 4.82) and high C-value (0.16–0.41, average = 0.33) indicate a short-term semihumid environment during Sha-1 Member (Figure 8B). In addition, as shown in the Ga/Rb–K_2_O/Al_2_O_3_ binary plot, all points in the low Ga/Rb ratios and intermediate K_2_O/Al_2_O_3_ ratio area suggest an abundance of illite and moderate weathering conditions associated with transitional semiarid and semihumid, cool and cold climatic conditions (Figure 8C). This result is confirmed by the binary diagram interpretation between SiO_2_ and (Al_2_O_3_ + K_2_O + Na_2_O) (Figure 8d), which indicates that Shaximiao Formation mudstone was deposited under a semihumid and semiarid climate (Figure 8D).

For CIA values are particularly sensitive to land surface temperature (LST), latitude of the lake basin, and soil depth in the drainage area, an experiential relationship between the CIA and LST as a paleothermometer was quantified as *T* (°C) = 0.56 × CIA – 25.7(*R*^2^ = 0.50), which is robust over a temperature range of 3–25 °C and has an uncertainty of approximately ±5 °C, corresponding to a CIA range of approximately 50–90.^53^ This method has been used to quantify the paleotemperature of land evolution in deep times.^54,55^ We use the relationship between CIA and LST to evaluate chemical weathering and temperature changes of Shaximiao Formation. The paleotemperature of Sha-1 Member ranges from 9.71 to 16.24 °C, with an average of 16.9 °C, and the value of Sha-2 Member is from 5.67 to 15.53 °C, with an average of 12.81 °C. These results are consistent with the paleotemperatures (7.9–17.4 °C) calculated by the PWI index and the SAL index based on paleosol outcrops.^16^ In summary, the paleoweathering indices and climatic indicators demonstrate that the studied samples of Shaximiao Formation experienced moderate chemical weathering and formed in semiarid to semihumid conditions. Moreover, paleotemperature *T*, C-value, and CIA index all suggest that the Sha-1 Member is wetter and warmer than the Sha-2 Member and experiences stronger weathering. These interpretations agree fairly well with association of mudstone lithofacies which is characterized by higher proportion of GGM, lower proportion of PRM, and wide occurrence of Estheria fossils in the shale interval at the top of the Sha-1 Member.

### 5.3 Paleosalinity in a Paleo-water Environment

**Figure 9:**
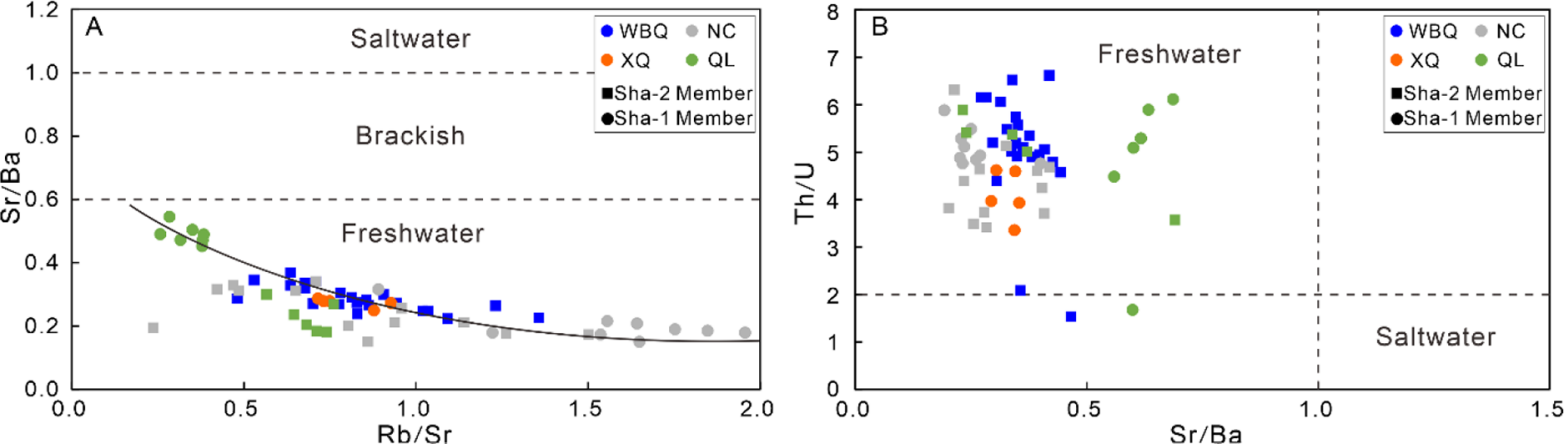
Analysis of paleo-water environment for the Shaximiao Formation mudstones. (A) Rb/Sr versus Sr/Ba discrimination diagram; (B) Sr/Ba versus Th/U discrimination diagram.

To ensure accurate determination of the paleosalinity of the paleowater body, we use multiple effective indexes to support paleosalinity interpretation. For Sr has higher solubility and migration ability in water than Ba, the Sr/Ba ratios of sediments usually indicate the salinity of the water body.^56,57^ The value of Sr/Ba in the Shaximiao Formation ranges from 0.16 to 0.55, with an average value of 0.29, which is far less than the critical value of freshwater (<0.6) (Figure 9A). K is mainly related to the content of clay minerals in mudstone, while Rb is more easily adsorbed by clay and organic matter, and its concentration increase with higher salinity. The ratio 1000 × Rb/K_2_O of less than 6 is used to represent freshwater.^30,58^ In this study, the value of 1000 × Rb/K_2_O in Sha-1 Member ranges from 1.77 to 4.39, with an average value of 3.70, and the value in Sha-2 Member ranges from 3.35 to 5.63, with an average value of 4.09, which indicates a freshwater environment with higher salinity than Sha-1 Member (Table 2). The Th/U ratio can also be used as a parameter to identify paleosalinity. In general, the Th/U ratio in freshwater environments is more than 2, and in saline water environments, it is less than 2 (Figure 9B).^3,11^ The Th/U ratio of the Shaximiao Formation is 1.54 to 8.68, with an average value of 5.08 and 4.95 for Sha-2 and Sha-1 Member, respectively (Table 2), indicating freshwater environments. To sum up, it is inferred that the Shaximiao Formation mudstone is mainly formed under a typical continental freshwater lake basin.

### 5.4 Paleoredox Conditions

**Figure 10:**
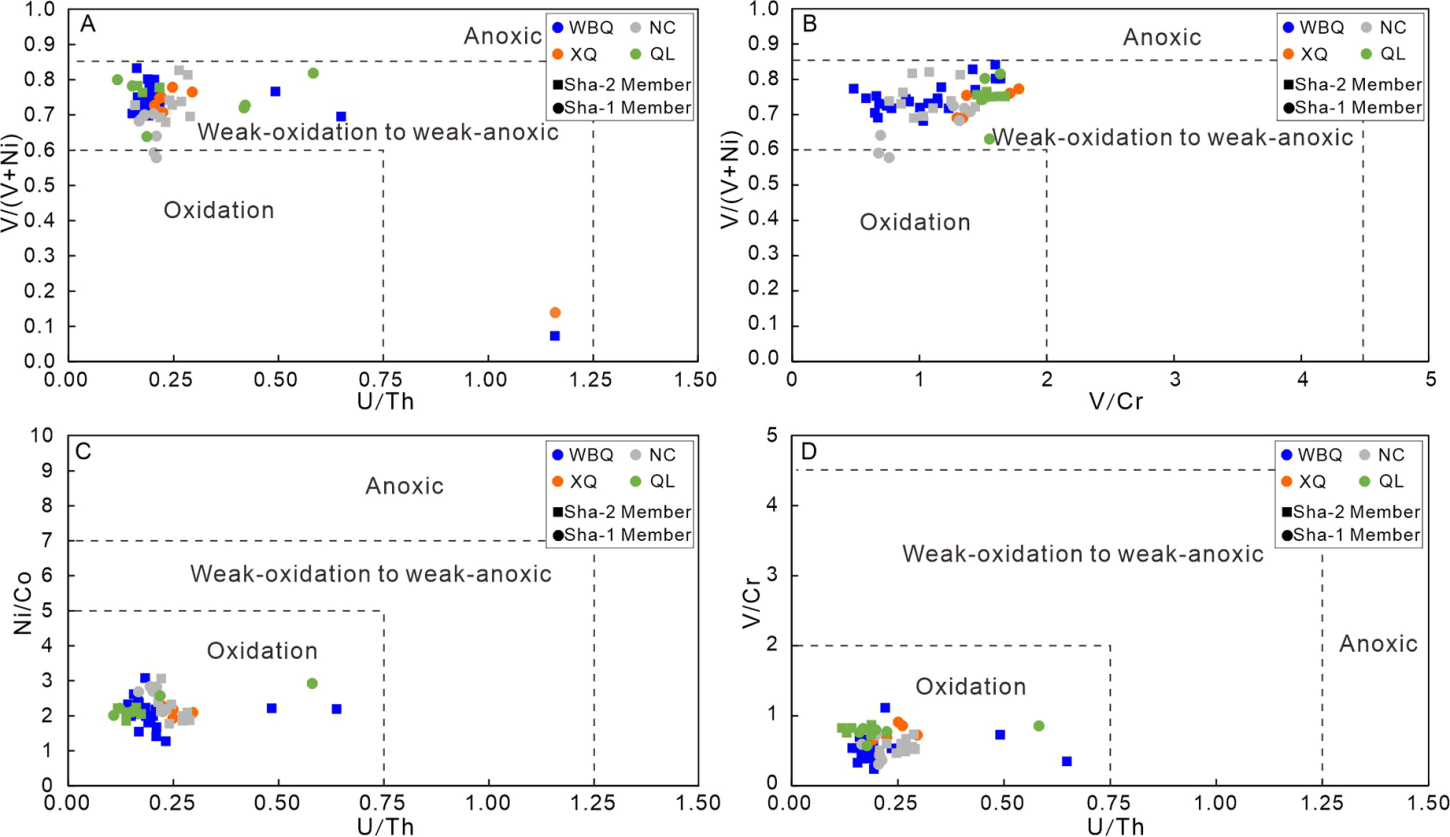
Analysis of paleoredox conditions for the Shaximiao Formation mudstones. (A) U/Th versus Ni/Co diagram; (B) U/Th versus V/Cr diagram; (C) U/Th versus V/(v + Ni) diagram; (D) V/Cr versus V/V + Ni) diagram.

V, Cr, U, Th, Co, Ni, and other oxidation-sensitive elements are easier to dissolve under oxidation conditions than under anoxic conditions.^1,59^ Therefore, they are used as good indicators to judge paleoredox conditions. According to previous studies, the redox state of the sedimentary environment can be characterized according to the ratios of V/Cr, V/(V + Ni), etc.^60,61^ The higher the V/(V + Ni) ratio, the lower the degree of oxidation of the water during deposition. It is widely accepted that the V/(V + Ni) ratio > 0.84, 0.54–0.82, and 0.46–0.6 represent anoxic, hypoxic, and oxygen-enriched sedimentary conditions, respectively (Figure 10A).^62^ The V/(V + Ni) range of Sha-1 Member is 0.58 to 0.82 (average = 0.71), and the value in Sha-2 Member varies from 0.68 to 0.87 (average = 0.75) (Table 2), indicating a hypoxic environment. The V/Cr value can also be used as a parameter to identify the redox environment. Generally, the V/Cr ratios >4.25, 2–4.25, and <2 indicate anoxic, dysoxic, and oxic conditions, respectively (Figure 10B).^63^ The V/Cr values of mudstone samples in Sha-1 Member range from 0.68 to 1.77, with an average of 1.33, and in Sha-2 Member, they vary from 0.48 to 2.21(average = 1.10), suggesting a weak to strong oxidation environment. The generally oxygen-enriched conditions are also supported by the widespread occurrence of purplish-red mudstone and the carbonate nodules of paleosols in the Shaximiao Formation.

In addition, the correlation between the Rb/Sr value and the Sr/Ba ratio (Figure 9A) shows a significant exponential correlation (*r* = 0.82), indicating that the local climate drought would lead to intense evaporation, reduce the lake level, and increase the salinity of the water body, resulting in a significant fluctuation of the Sr/Ba and Th/U values. However these processes do not significantly change the paleoenvironment of the Sichuan Basin during deposition of Shaximiao Formation, resulting in a relative stable lake water body dominated with freshwater and oxidation-weak oxidation conditions.

### 5.5 Reconstruction of the Paleoenvironmental Model

The sediment sources in the Shaximiao Formation in the Sichuan Basin are mainly affected by Dabashan and Micangshan followed by Longmenshan in the west and Xuefengshan in the southeast, resulting in the distribution of the sedimentary system in the direction of NE–SW.^21,64^ The sedimentary environment elements, which include meandering rivers, lakes, and paleosols, are identified by outcrop and core evidences.^18^ The stratigraphic sequence shows a high ratio of mudstone to sandstone. The fine-grained sediments are dominated with meandering river mudstones and lake mudstones intercalated with paleosol intervals. The seismic profiles in central Sichuan show imbricated foreset reflections, representing the progradation of river-delta systems into the shallow water environment, feeding the lake basin with clastic materials derived from Dabashan, Micangshan, Longmenshan, and Xuefengshan.^65,66^ Therefore, the subenvironment of Shaximiao Formation can be divided into alluvial fan, river, delta, and lake from the source to sink.

**Figure 11:**
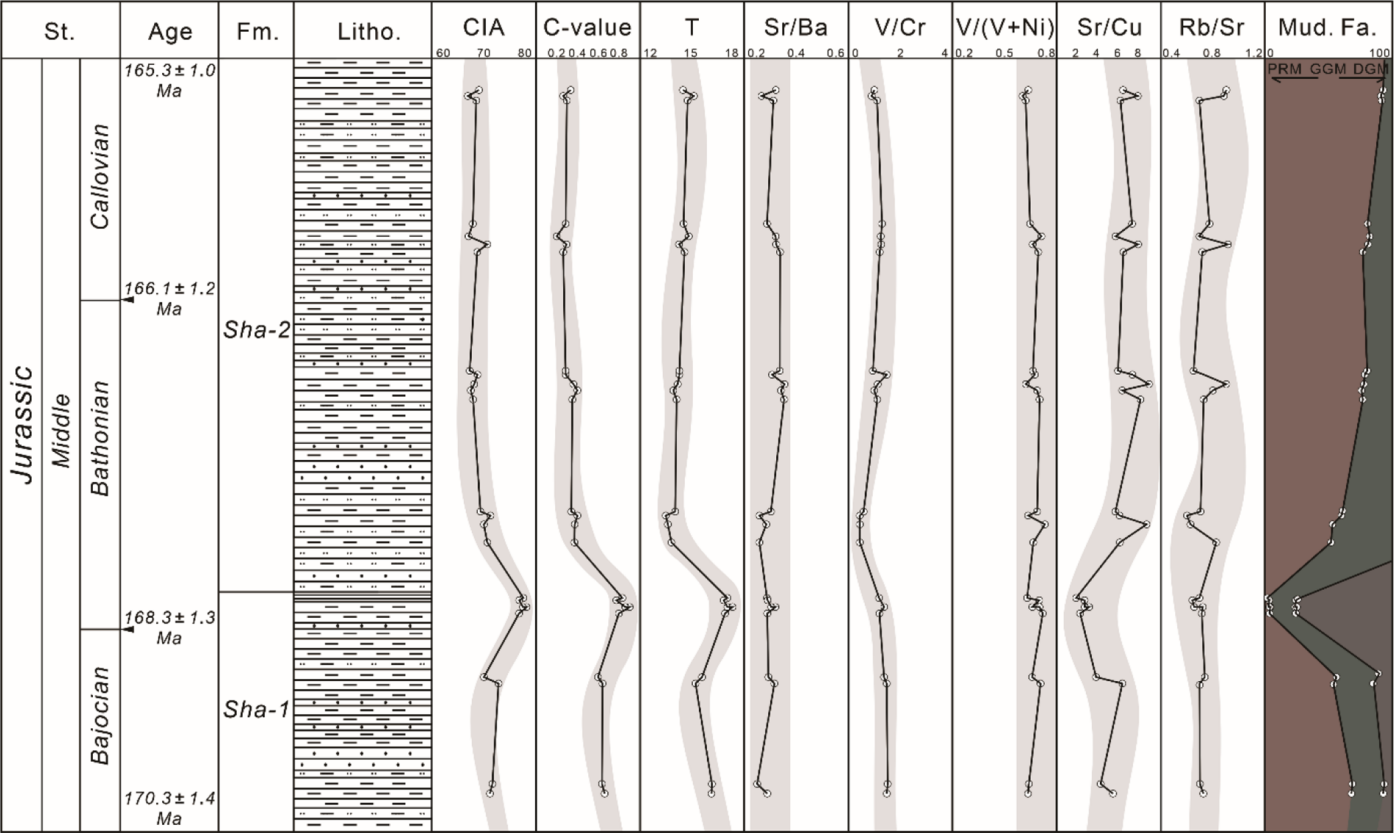
Variations of different elemental geochemical parameters in the Shaximiao Formation.

The long-term similarity and short-term local differences in the evolution history of geochemical indicators in the Shaximiao Formation are evident. A subhumid to semi-arid climate with strong seasonal aridity is interpreted by the absence of lignite and carbonaceous plant debris and the abundance of purple-red mudstones that developed under a lowered water table condition. As interpreted to be deposited in a weak-oxidation environment (indicated by paleoredox indices, Figure 10) during a relative humid climate (indicated by C-value and paleotemperature *T*, figure 8) with relative intense chemical weathering (indicated by paleo-weathering indices, Figure 8), the dark-gray mudstone/shale interval on upper part of Sha-1 Member is speculated to be deposited in a shallow water lake during a short-term humidity phase. It is concluded that although semiarid and semihumid climates dominate the Shaximiao Formation stage, the sedimentary basin would experience a short-term humidity phase in the late stage of Sha Member characterized by shallow water depth (Figure 11).

**Figure 12:**
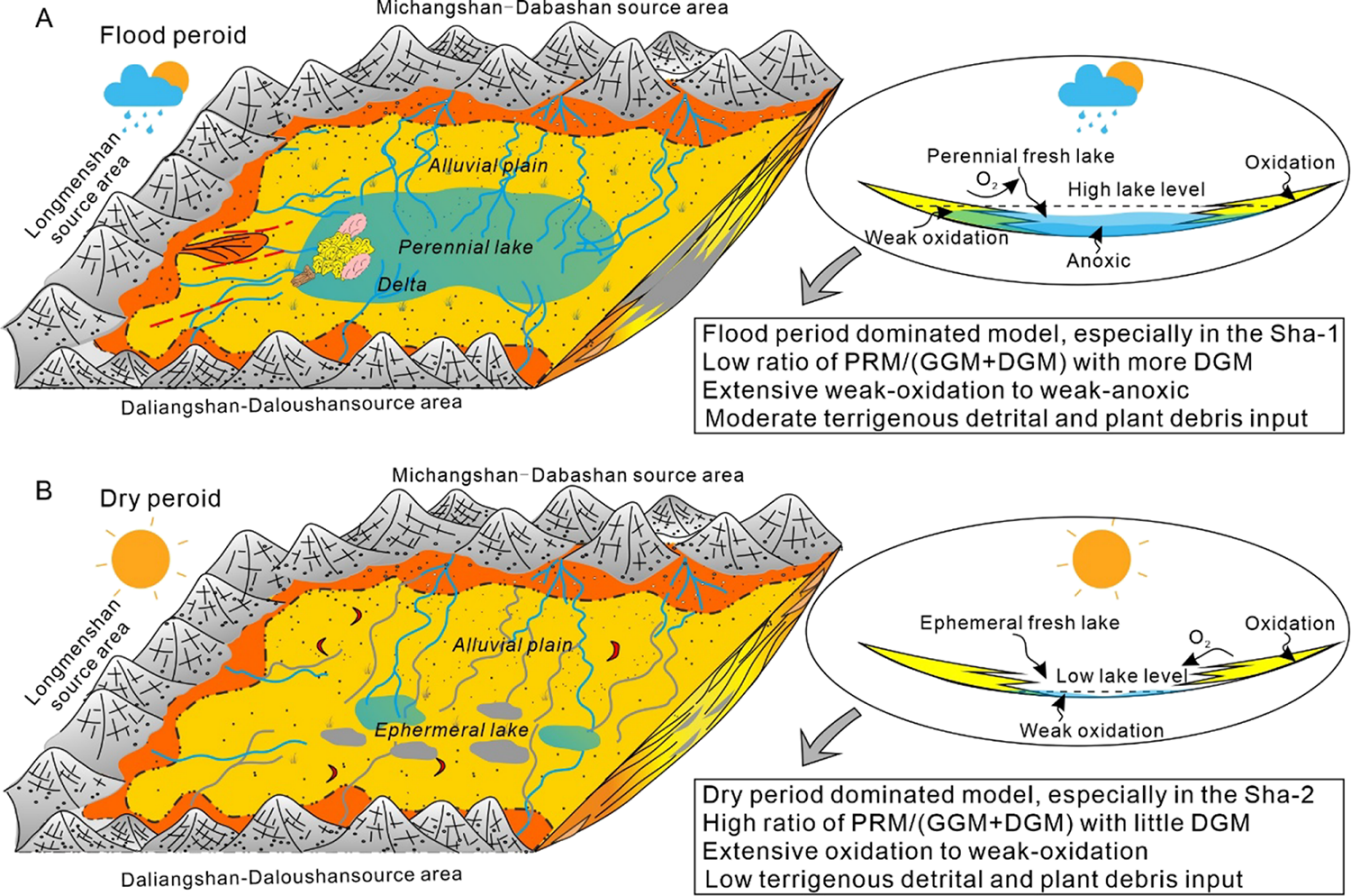
Proposed conceptual model shows the paleoclimatic and paleoenvironmental conditions. (A) Alluvial plain - perennial lake model at flood period; (B) alluvial plain - ephemeral lake model at dry period.

Based on the characteristics of mudstone sedimentology and element geochemistry, a sedimentary environmental model is proposed for the Shaximiao Formation, including two stages: (1) the alluvial plain-perennial lake stage at flood/wet periods and (2) the alluvial plain-ephemeral lake stage at dry periods. During the flood/wet period, due to the abundant seasonal precipitation, the riverine discharge and sediment budget increase significantly, resulting in the rapid expansion of the lake area, the formation of a unified large lake in center of the basin, and widely distribution of delta and lacustrine deposits, with limited sourceward retreated alluvial plain (Figure 12A). This period is characterized by stronger chemical weathering and a higher sedimentation rate. Given the “short term” attribute of wet period and shallow water depth, extensive reduction conditions would not develop, and most of the detrital material was preserved in weak oxidation to weak-anoxic environment. DGM and GGM are the dominated mudstone lithofacies in this stage, with PRM distributed at the margin of the basin. This stage exists mainly for Sha-1 Member. Under the long-term semiarid and semihumid climate, Shaximiao Formation is dominated with the alluvial plain-ephemeral lake stage, especially in the Sha-2 Member (Figure 12B). During this dry stage, for rare precipitation and intense evaporation, the lake experiences low levels with the extensive basin floor having been exposed as alluvial/lake plain. The alluvial/lake plain deposits, which include channel sandstones and floodplain mudstones, dominate the stratigraphic record. During this stage, the sedimentary basin which is characterized by weak chemical weathering and strong oxidation, is dominated by sporadic small lakes/playas, ephemeral rivers, and discontinuous paleosols. PRM, distinctive soil horizons, carbonate nodules, and root traces are the main indicators of this stage. The sediments in Sha-2 Member mainly deposited in this stage, although it also contains interval developed from flood/wet stage.

### 5.6 Correlation of Regional Climate in Middle Jurassic

Global geodynamics during the Middle Jurassic may have exerted an influence on climatic fluctuations, thus shaping the alluvial plain-lake environment model of the Shaximiao Formation. The Middle Jurassic global climate was characterized by zonal climate characteristics of arid, tropical, northern tropical, boreotropical, warm temperate, and cold temperate environmental conditions from the equator to the poles.^67^ The Jurassic is a typical greenhouse climate period, with long-term climate changes and short-term fluctuations recorded in continental basins.^68,69^ Plate tectonics is the key driving force for shaping paleoclimate and paleogeography.^70^ The climate of the depositional basin is generally considered to be closely related to paleogeography. As the supercontinent Pangea split apart, intense plate tectonic movement led to significant volcanic activity, mountain-building events, and the attachment of islands onto continents during the Jurassic,^71,72^ resulting in reorganization of paleogeographic and paleoclimatic zones of the Sichuan Basin.^73^ During the Middle Jurassic, been interpreted to be located between 20 °N and 30 °N in the northern tropical climate zone,^74^ the paleotemperatures of the Sichuan Basin are supposed to be ∼20 for geochemical evidence suggests that surface waters in the low latitudes were ∼20 °C (68 °F). The assumption is confirmed by temperature calculated from the relationship between weathering index and LST during the Shaximiao Formation (ranging from 11.6 to 17.7 °C) and calculated from the salinization (SAL) index (ranging from 10 ± 4.4 °C to 13 ± 4.4 °C).^16^

Middle Jurassic sedimentary sequences, which are dominated with red sediments deposited in similar paleoclimates and paleoenvironments, have been discovered in many sedimentary basins in the Northern Hemisphere including the Junggar Basin, Qaidam Basin, and Santanghu Basin. In the Junggar Basin, the sedimentary environment changes from deltaic to fluvial depositional systems with enhanced aridity.^75^ In the Qaidam Basin, an upward weakening and even arrested coal accumulation caused by dry and/or arid episodes have been confirmed by the high diversity of spores and pollen grains. A sudden decrease of the chemical weathering index, which reveals a rapidly transformed climatic state from humid to seasonally arid also been revealed in the Aalenian-Bajocian of the Santanghu Basin.^76^ These strata records from different sedimentary basins of the Northern Hemisphere may be related to a transient global warming phenomenon or a strong greenhouse effect^77^ as well as Shaximiao Formation in the Sichuan Basin. Global wildfires have probably consumed enormous amounts of O_2_ from the atmosphere, and huge amounts of greenhouse gases were released, inducing the Jurassic global warming event, with palaeoatmospheric CO_2_ four times higher than today in the Early Mesozoic period.^78^ Estimated pCO_2_ fluctuations in the Sichuan basin coincide with paleotemperature variations in the low paleolatitudes of the Northern Hemisphere. In addition, according to the seaway dynamics model, which emphasizes the potential significance of the seaway for heat transport, major modifications in Mesozoic oceanic current patterns may have a significant influence on a large-scale, abrupt climate change and potentially govern transformations between warm/cool or dry/humid climate modes.^79^ As Korte (2015) proposed, the uplift of the North Sea Dome that impeded northward oceanic heat transport was considered to cause an especially abrupt mid-latitude cooling of seawater by as much as 10 °C in the north–south Laurasian Seaway during the Jurassic.^80^ Interestingly, Shaximiao Formation shows an earlier climate change from humid to seasonally arid during Middle Jurassic indicated by small cumulative thickness of dark-gray shale and deficiency of a coal bed comparing with equivalent stratigraphic records in the Junggar Basin, Qaidam Basin, and Santanghu Basin. The lithological difference of Shaximiao Formation probably due to reorganization of paleoclimate zones of Sichuan Basin beyond orogen belts along plate sutures (e.g., Qinling orogen). As the lee side (or rain shadow) of the Qinling Orogen and Longmenshan Orogen, the Sichuan Basin may receive considerably less precipitation as Tethys trade winds zone.

In summary, possibly having been controlled by global geological events and local topography, the paleoclimate of Middle Jurassic Shaximiao Formation in the Sichuan Basin is primarily semiarid and semihumid, with Sha-2 Member precipitated in a drier, cooler, and more oxygen-rich environment.

## 6 Conclusions

Combined with the analysis of element geochemistry and sedimentology of mudstone, different types of mudstone lithofacies are identified, the characteristics of the paleoclimate, paleowater body, and paleooxidation environments are recovered, and a sedimentary environmental model including wet and dry stages is proposed for Middle Jurassic Shaximiao Formation in the Sichuan Basin.

(1) Three mudstone lithofacies can be identified based on color including purplish-red mudstone (PRM), gray-green mudstone (GGM), and dark-gray mudstone (DGM), with DGM exists only in the Sha-1 Member, and the proportion of PRM gradually increases from Sha-1 to Sha-2 Member.
(2) The geochemical characteristics of mudstone indicate that the main source of sediments are felsic and intermediate igneous rocks derived from passive continental margin.
(3) The Shaximiao Formation mudstones, which suffered moderate chemical weathering, are mainly deposited in a shallow oxygen-rich freshwater lake basin under semiarid–semihumid, cool-cold climatic conditions, with Sha-2 Member precipitated in a drier, cooler, and more oxygen-rich environment. The regional paleoclimate is possibly controlled by global geological events and local topography.
(4) A two-stage sedimentary environmental model is proposed for the Shaximiao Formation. The wet stage environment is dominated by a large perennial lake surrounded by limited alluvial plain, and the dry stage environment is characterized by small ephemeral lakes scattering in extensive alluvial plain.
